# Regulatory significance of terminator: A systematic approach for dissecting terminator-mediated enhancement of upstream mRNA stability

**DOI:** 10.1016/j.synbio.2024.11.006

**Published:** 2024-11-28

**Authors:** Jia-Wei Ren, Jin-Peng Zhang, Zi-Lun Mei, Jia-Yi Shao, Guo-Qiang Xu, Hui Li, Jin-Song Gong, Xiao-Mei Zhang, Jin-Song Shi, Xiao-juan Zhang, Zheng-hong Xu

**Affiliations:** a School of Biotechnology and Key Laboratory of Industrial Biotechnology of Ministry of Education, Jiangnan University, Wuxi, 214122, PR China; b National Engineering Research Center for Cereal Fermentation and Food Biomanufacturing, Jiangnan University, Wuxi, PR China; c School of Life Science and Health Engineering, Jiangnan University, Wuxi, 214122, PR China; d Innovation Center for Advanced Brewing Science and Technology, College of Biomass Science and Engineering, Sichuan University, 24 Southern Yihuan, Chengdu, 610065, PR China

**Keywords:** Terminator, Termination efficiency, Transcription, Upstream mRNA stability

## Abstract

The primary function of terminators is to terminate transcription in gene expression. Although some studies have suggested that terminators also contribute positively to upstream gene expression, the extent and underlying mechanism of this effect remain largely unexplored. Here, the correlation between terminating strength and upstream mRNA stability was investigated by constructing a terminator mutation library through randomizing 5 nucleotides, assisted by FlowSeq technology, terminator variants were categorized based on the downstream fluorescence intensity, followed by high-throughput sequencing. To examine the impact of terminators on mRNA stability, the abundance of downstream gene transcripts for each terminator variant was quantified through cDNA sequencing. The results revealed that the transcript abundance controlled by strong terminators was, on average 2.2 times greater than those controlled by weak terminators on average. Moreover, several distinct features could be ascribed to high relative abundance of upstream gene transcript, including a high GC content at the base region of hairpin, and a high AT content in downstream of the U-tract. Additionally, these terminators showed a free energy between −28 and −22 kcal/mol, and a stem length of 14 nt. Finally, these features ascribed the upstream beneficial terminator were validated across various expression systems. By incorporating the optimal terminator downstream of RSF, GSH and HIS in three different strains, the fermentation productions-NMN SAM and VD13 exhibited a remarkable enhancement of 30 %–70 %. The findings presented here uncovered the terminator characteristics contributed to the upstream mRNA stability, providing guiding principles for gene circuit design.

## Graphical abstract

**Figure:**
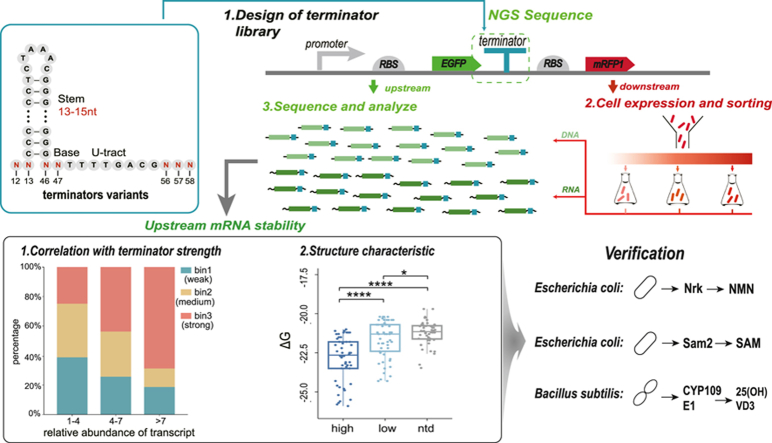

## 1 Introduction

Gene expression regulation involves two crucial processes: transcription and translation [[1], [2], [3]]. At each of these two levels, different biological components play regulatory roles [[4], [5], [6], [7], [8], [9]]. In prokaryotes, transcription initiates when RNA polymerase identifies the promoter region and concludes when it identifies the terminator [10,11]. Currently, a set of influential studies investigated the process of transcriptional termination controlled by terminators [[12], [13], [14]]. There are two main forms of transcription termination: ρ-factor-dependent terminators terminate the transcriptional process with the joint participation of the ρ-protein and the cofactor NusG; whereas intrinsic terminators rely on their secondary structure to perform the function of terminating transcription. The process of transcription termination by intrinsic terminators occurs at the GC base-rich stem-loop, the 3′ end of the stem-loop structure is a U-tract structure, and the A-tract structure often appears at the upstream of the hairpin which is usually related to the encoding of a bidirectional intrinsic terminator [[15], [16], [17], [18]]. In contrast to the complex regulation of the ρ-factor-dependent transcription termination mechanism, intrinsic terminators, which rely on the self-folding of their sequences to form a hairpin structure, offer more straightforward and easily regulated tools for gene expression regulation [[19], [20], [21], [22], [23], [24]].

The primary function of a terminator is to halt the transcription process of an upstream gene, thereby preventing transcriptional read-through [25]. Transcriptional read-through hampers RNA polymerase recycling and imposes an additional burden on overall transcription efficiency [[26], [27], [28], [29]], consequently impacting downstream gene expression. Consequently, investigating the factors influencing terminator efficiency had been a focal point in terminator research. Ahn et al. demonstrated that the termination efficiency is influenced by the length of the stem in the hairpin structure of the terminator [30]. Chen et al. [31] designed an *E. coli* terminator library capable of holding up to 582 entries and discovered that the stem structure of the strong terminator had a higher GC content, while no similar pattern was observed near the ring structure. Cui et al. conducted a study to assess the termination efficiency of 80 natural terminators in *Bacillus subtilis* [32]. They discovered that the termination efficiency of the terminators was highly correlated with the U content in the U-tract of the terminator. The termination efficiency of the terminator was influenced not only by the terminator sequence itself but also by the surrounding upstream and downstream contexts. Moreover, evidence demonstrates that the termination efficiency of the terminator is influenced by the length of the spacer sequence located between the terminator and the upstream gene [33]. Olivier et al. found that changing the composition of bases downstream of the terminator impacted the efficiency of the terminator [34]. Recently, our group identified three parameters describing the detailed characteristics of terminators regarding their muti-functional roles in gene transcription and translation, including transcription shut-down degree (α) and upstream mRNA protection capacity (β), and apparent termination efficiency (η) reflecting the overall regulatory effect of the terminator. Furthermore, the factors that influence downstream gene termination efficiency were characterized based on the library of terminator mutations. It was found that terminators consisting of a hairpin with low free energy and a flawless U-tract structure were more effective in shutting down downstream gene expression [35,36].

Beside of cessation of the downstream gene transcription, emerging evidence suggests that terminators also exert an impact on upstream gene expression. Mairfofer J et al. utilized a combination of synthetic terminators to enhance termination efficiency up to 99 %, and the upstream gene protein expression was also effectively improved [37]. In eukaryotes, the terminator consists of a 3′-UTR untranslated region and a region that terminates mRNA transcription, can prevent the hydrolysis of intracellular enzymes thereby protecting the mRNA of the upstream genes from degradation [38,39], and this protective effect is closely related to the 3′-UTR region, and the features including the content of the GC bases within the 3′-UTR, the length of the region, and the secondary structure ascribed to the upstream gene mRNA stability. However, in prokaryotes, the underlying mechanism by which terminators enhance upstream gene expression has not been thoroughly investigated.

mRNA degradation is one of the crucial determinants of gene expression. There exist various hydrolases involved in this process, including endonucleases (such as RNases E, G, III), exonucleases (such as RNase II, PNPase, RNase R), and coenzymes that modify mRNA terminal coenzymes (such as *E.coli* RppH, DapF, RppH, RppH) [40]. Studies have demonstrated that terminators can maintain mRNA stability by obstructing mRNA cleavage by RNase hydrolase [11,41]. Correlative investigations in *Lactococcus lactis* and *Saccharomyces cerevisiae* have further confirmed the beneficial impact of terminators in prolonging mRNA survival rate and safeguarding mRNA [[42], [43], [44], [45]]. Nevertheless, the specific attributes of terminators that contribute to the optimal preservation of mRNA have not been well examined. Therefore, further investigation into the intricate interplay among terminator strength, secondary structure, and mRNA stability is imperative for the rational design of terminators with optimal performance.

In this study, we constructed a terminator mutation library utilizing T7 terminator as a template, with partially randomization at positions M12-M13, M46-M47, and M56-M58. Subsequently, FlowSeq technology was employed to categorize terminator variants based on the downstream fluorescence intensity followed by high-throughput sequencing. The corresponding abundance of upstream gene transcript regulated by each terminator variant was also quantified by sequencing of cDNA, thereby attributing terminator characteristics associated with enhanced mRNA stability. The terminators with strong protective effects on mRNA in different genetic contexts. This was achieved by assessing the gene expression of *nrk*, *sam2*, and *cyp109e1*, as well as the production of corresponding fermentation products (NMN, SAM, and 25(OH)VD3) in *Escherichia coli* and *Bacillus subtilis*. These findings have practical implications for the rational design of terminators as enhancers of mRNA stability.

## 2 Materials and methods

### 2.1 Bacterial strains and growth conditions

*E. coli* BL21 (DE3) was used as the host bacterium, and the plasmids were constructed. The strains were cultured in LB medium (peptone 10.0 g/L, yeast powder 5.0 g/L, NaCl 10.0 g/L) at 37 °C. When solid medium was required, 2 % agar powder was added to the above medium. The concentration of antibiotics was: kanamycin 50.0 mg/L. When a reporter gene was required for expression, 0.5 mmol/L isopropyl β-D-thiogalactoside (IPTG) was added.

### 2.2 Construction of terminator mutation libraries

The template DNA of the terminator mutation library was divided into conserved and mutated regions, and was first annealed by diluting the dry powder of the corresponding forward and reverse strand primers to 20 μM, and mixing 10 μL of each in a PCR instrument, and then carrying out the reaction as follows: 95 °C for 5 min, 94 °C for 1 min, 93 °C for 1 min, 92 °C for 1 min, 91 °C for 1 min, and 90 °C for 1 min. At the end of the reaction, the samples were immediately placed in boiling water and cooled to room temperature, at which time the second-strand RNA fragment complementary to the conserved region of the template DNA strand was annealed to the template DNA. The second strand of RNA was then amplified by Klenow enzyme (NEB, the USA) and flattened to flat ends by incubation at 25 °C for 15 min, followed by the addition of EDTA at a final concentration of 10 mM and reaction at 75 °C for 20 min to remove the enzyme activity. Finally, the finished sample was placed at −20 °C for later use. T4 polyphosphorylase (NEB, the USA) was used to phosphorylate the 5′-end of the sample formed by the above reaction for the next step of the ligation reaction. The linearized plasmid was acquired by double digestion with restriction enzymes *Hin*d III and *Eco*R I, and then the linearized plasmid was linked with the fragment overnight at 4 °C using T4 DNA ligase. The ligation product was distributed into 10 tubes of *E. coli* BL21 sensory cells. After being heated to 42 °C, LB medium was added to each tube to reach a final volume of 1 mL. Following incubation at a temperature of 37 °C for 1 h at 220 rpm, all of the bacterial fluids were transferred and combined with 100 mL of LB liquid medium supplemented with kanamycin (50 mg/L) for overnight incubation.

### 2.3 Screening mutant libraries using flow cytometry

Two milliliters of the mutant library was added to 100 mL of LB liquid medium containing kanamycin. Isopropyl β-d-1-thiogalactopyranoside (IPTG) was then added to a final concentration of 0.5 mM to induce protein expression. The culture was incubated at 37 °C for 2 h. Subsequently, the bacteria were screened by flow cytometer cytometry. The Becton Dickinson FACSAria III flow cytometer was set to detect mRFP1 fluorescence at an emission wavelength of 561 nm–610/20 nm. Data from the flow cytometer were analyzed using FlowJo v10 software to calculate the average fluorescence intensity. Then cells were classified according to the intensity of downstream red fluorescence into weak terminators (bin1), medium terminators (bin2) and strong terminators (bin3).

### 2.4 High-throughput sequencing

The collected cells in the 3 groups were inoculated into 100 mL LB medium supplemented with kanamycin, cultured with 220 rpm shaking at 37 °C for 12 h, the plasmid was extracted by taking 1.5 ml of the bacterial solution and the DNA amplification product was obtained by PCR amplification using the target primers. We then extracted RNA from the bacterial broth using Total RNA Isolation Kit (Vazyme Bio Inc) and reverse transcribed the RNA to cDNA using cDNA Synthesis Kit (Vazyme Bio Inc), followed by PCR amplification using the target primers to obtain the RNA amplified fragments. Finally, 1000 ng of DNA and RNA amplified fragments from each of the three groups were taken for high-throughput sequencing.

### 2.5 Data analysis

The process of extracting the terminator sequences from the fastq file is shown below. The contig sequences were excised using cutadapt (1.2.1) software, and quality control was performed using prinseqlite (0.19.5) software to ensure that the quality value of each base was greater than 30. Seqkit (0.10.1) software was used to complete the sequence extraction task. Also, sequences with at least one T in the first three bases of the U-tract site were considered termination sequences and were retained. The RNA fold and RNA eval software in the Vienna RNA package (2.5.0) toolkit were utilized to predict the secondary structure and to obtain the free energy of each terminator sequence ΔG.

### 2.6 Calculation of transcript levels

By comparing RNA reads to DNA reads, it is possible to determine the amount of DNA that has been translated into RNA, allowing for the calculation of the change in transcript levels. We will analyze transcript levels by considering both transcript abundance and the relative abundance of transcript, which will be determined as follows:

$$\text{transcript}\;\text{abundance}=\frac{RNA\;reads}{DNA\;reads}$$

$$\text{relative}\;\text{abundance}\;\text{of}\;\text{transcript}=\frac{\frac{RNA\;reads}{total\;RNA\;reads}}{\frac{DNA\;reads}{total\;DNA\;reads}}$$

### 2.7 Determination of mRNA survival rate

Single bacteria were picked and inoculated into 10 mL of LB liquid medium supplemented with kanamycin and incubated at 37 °C, 220 rpm for 12 h, followed by taking 200 μL and adding it to 10 mL of LB liquid medium supplemented with kanamycin and incubating at 37 °C, 220 rpm for 1.5 h until the OD was between 0.4 and 0.6. Added IPTG with a final concentration of 0.5 mM and Rifamycin with a final concentration of 500 μg/mL, incubated at 37 °C, 220 rpm for 2 h, and took 1 mLof the bacterial solution as a 0min sample, and then put the remaining bacterial solution at 37 °C, 220 rpm environment to incubate for 15 min, and took 1 mL of the bacterial solution as a 15 min sample. RNA was extracted from 1 mL of bacterial fluid taken at both time points using Total RNA Isolation Kit (Vazyme Bio Inc), followed by reverse transcription of RNA to cDNA using cDNA Synthesis Kit (Vazyme Bio Inc), the Quantitative Real-time PCR (qPCR) were followed, and calculation of half-life according to the formula:

$$\Delta C_{q,x}=C_{q,x}-C_{q,r}$$

$$mRNA\;survival\;rate=2^{-\left(\Delta C_{q,x,15\;\min}-\Delta C_{q,x,0\;\min}\right)}$$

Where x denotes the sample, $C_{q,x}$ is the quantization period of its target gene, $C_{q,r}$ is the quantization period of its internal reference gene. $C_{q,x,15\;\min}$ denotes the sample that reacted for 15 min after the addition of rifamycin (1 ‰), and $C_{q,x,0\;\min}$ denotes the sample to which rifamycin was just added.

### 2.8 Determination of shake flask fermentation yield of β-nicotinamide mononucleotide

Single bacteria were picked and inoculated into 10 mL of LB liquid medium supplemented with kanamycin and incubated at 37 °C, 220 rpm for 12 h, followed by taking 600 μL and adding it to 30 mL of LB liquid medium supplemented with kanamycin and incubating at 37 °C, 220 rpm for 2 h until the OD was between 0.6 and 0.8. Add the final concentration of 0.01 mM IPTG, 25 °C, 220 rpm fermentation 12 h, take 2 mL of fermentation solution washed (Tris hydrochloric acid) twice, cast the substrate at 37 °C reaction for 2 h, add 20 % perchloric acid to terminate the reaction, centrifuged at 12,000 rpm for 10 min, the supernatant was taken through the aqueous membrane, and the production was determined using HPLC. Three biological replicates were conducted here.

### 2.9 Determination of shake flask fermentation yield of S-adenosyl-l-methionine

Single bacteria were picked and inoculated into 10 mL of LB liquid medium supplemented with kanamycin and incubated at 37 °C, 220 rpm for 12 h, followed by taking 1 mL and adding it to 550 mL of LB liquid medium supplemented with kanamycin and incubating at 37 °C, 220 rpm for 2 h until the OD was between 0.4 and 0.6. Add the final concentration of 0.3 mM IPTG, 25 °C, 220 rpm fermentation 12 h, The substrate was cast and reacted at 25 °C for 2 h. 1 mL of fermentation broth was taken and centrifuged at 12,000 rpm for 10 min, and the supernatant was taken over an aqueous membrane and the yield was determined using HPLC. Three biological replicates were conducted.

### 2.10 Determination of shake flask fermentation yield of 25(OH)VD3

Single bacteria were inoculated into 10 mL TB vials (1 ‰ kana) and incubated at 37 °C, 220 rpm for 12 h, followed by transferring 1 mL–50 mL TB vials (1 ‰ kana), adding substrate, and incubating at 37 °C, 220 rpm for 36h. 1 mL of fermented bacterial broth was added with 700 μL of ethyl acetate, and incubated at 2700 rpm for 4 min, followed by centrifugation at 12000 rpm for 10 min. Then the supernatant was put into a fume hood to evaporate overnight, and 200 μL was added to re-solubilize the supernatant, which was then passed through an organic membrane and the concentration was determined by HPLC. Three biological replicates were conducted.

### 2.11 The measurement of the mRNA level

One milliliter of cells after fermentation culture was removed and RNA was extracted from the samples using FastPure Cell/Tissue Total RNA Isolation Kit V2 (Vazyme). CDNA was then synthesized using random primers by HiScript III All-in-one RT SuperMix Perfect for qPCR reagent (Vazyme) to reverse transcribe and synthesize single-stranded cDNA. custom 16S rRNA primers were used as intrinsic control genes, and Quantitative Real-time PCR (qPCR) was performed according to the protocol of the TB Green® Rapid qPCR Mix (TAKARA Bio Inc). The mRNA levels were calculated as follows:

$$\Delta C_{q,x}=C_{q,x}-C_{q,r}$$

$$\Delta C_{q,cb}=C_{q,cb}-C_{q,cr}$$

$$\Delta \Delta C_{q}=\Delta C_{q,x}-\Delta C_{q,cb}$$

$$mRNA\;level=2^{-\Delta \Delta C_{q}}$$

Where x denotes the sample, r denotes the internal reference gene 16sRNA. $C_{q,x}$ is the cycle period of its target gene, is the cycle period of its internal reference gene. And cb is the control sample, $C_{q,cb}$ is the quantization period of its quantization, and $C_{q,cr}$ is the quantization period of its internal reference gene.

## 3 Results and discussion

### 3.1 Terminators with varied terminating strength affecting upstream mRNA stability

**Fig. 1:**
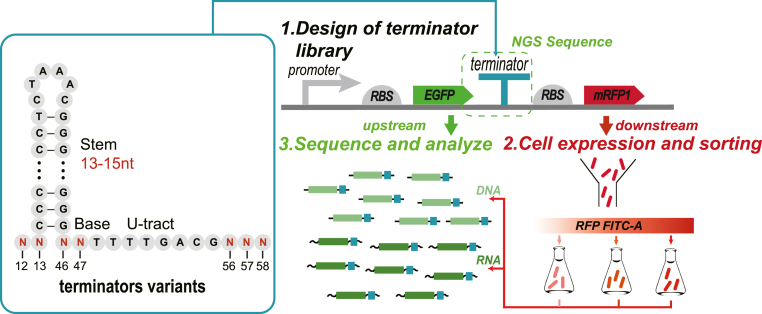
The principal scheme of the FlowSeq experiment. Presented are the steps of terminator mutation library design and transformation, as well as cell sorting and sequencing via FlowSeq techniques to obtain the phenotype and genotype characteristics of terminator variants. The Red letters represent mutated bases, numbers represent base positions. (N: A/T/C/G).

A strong terminator T7 was used as a template, and M12-M13, M46-M47, and M56-M58 positions were partially randomized to create a terminator mutation library (Fig. 1). All terminator variants in this study were predicted to be capable of forming a 13-15 nt stem and 6 nt loop structure. The constructed terminator library was transformed and expressed in *Escherichia coli* BL21(DE3) and the cells were further sorted by flow cytometric. Terminator strength refers to the ability of the terminator to terminate the transcription to avoid read-through of downstream genes. Strong terminators can terminate the transcription more efficiently, and the corresponding leaky transcriptional read-through of downstream genes is weaker. On the contrary, weak terminators lead to higher leakage of downstream gene expression. Therefore, based on the downstream gene expression differences (mRFP1 fluorescence intensity), cells with varied terminators were sorted into three bins, including bin1 (weak terminators), bin2 (medium terminators) and bin3 (strong terminators).

**Fig. 2:**
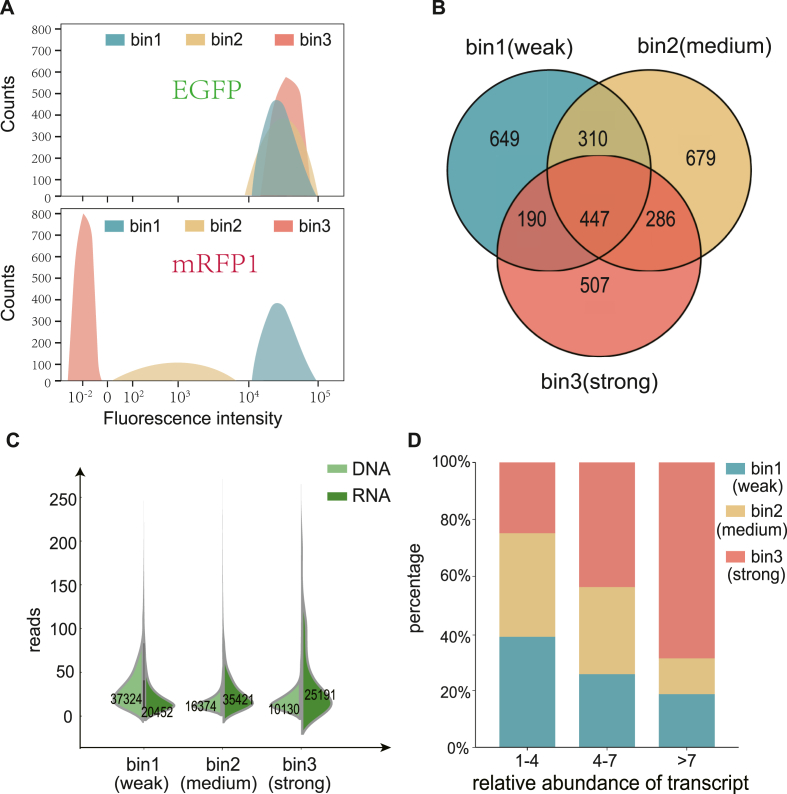
Effect of weak, medium, and strong terminators on the abundance of upstream gene transcript. A. The fluorescence distribution of the upstream and downstream reporter genes was analyzed in each bin, and cells were sorted based on their mRFP1 (downstream gene) expression. B. A Venn diagram illustrating the distribution of terminator sequences across three bins was constructed, excluding sequences with a detection of only 1–2 reads. C. Comparison of DNA and RNA reads in three bins. D. The relative abundance of transcript regulated by terminators with varied strengths was compared. The dataset used for C and D excluded overlapping sequences among bins.

Among them, the highest level of red fluorescence under the regulation of the terminator was observed in bin1, followed by bin2, and the lowest in bin3. The specific information in each bin was presented in Fig. 2A. To observe the variations in transcript quantity of upstream gene expression regulated by each terminator variant, high-throughput sequencing was conducted on both DNA and RNA of terminator sequences in each collected bin [46]. The sequences generated by high-throughput sequencing were further screened according to several criteria, including 1) as the most basic sequence characteristics of terminators summarized by Lesnik et al., the beginning 3 bases in front of the U tract must possess one U base [47]. There are approximately 7000–8000 terminator sequences each bin included in accordance with the characteristic; 2) Sequences with only one or two reads detected in any bin were excluded to minimize the impact of accidental events (Fig. 2B). 3) Additionally, overlapping sequences among bins were eliminated, and only the sequences with more than 3 reads detected and exclusively present in a specific bin were considered.

To investigate the variation in transcript abundance of upstream genes under the regulation of terminators with different terminating strength, the total number of DNA and RNA reads in each bin were calculated (Fig. 2C). There were significantly more DNA reads than RNA reads in bin1 (weak terminators). In contrast, bin 2 (medium terminators) had significantly fewer DNA reads compared to RNA reads, indicating that more transcripts were obtained bin 2 compared with that of bin 1. This discrepancy of DNA and RNA reads was even more noticeable in bin 3 (strong terminators). The average RNA reads of bin 3 reached 3.2 times of that in bin 1. These results revealed that although the strength of a terminator determines the ability to shut down the leaking transcription of downstream gene, the abundance of upstream gene transcripts increased with the higher terminator strength as well.

In order to eliminate any bias due to differences in the sampling fractions (bins), the number of reads of each sequence was normalized. Specifically, the relative abundance of transcript regulated by each terminator variants was calculated by the ratio of corresponding normalized number of RNA reads to normalized number of DNA reads within each bin. A higher relative abundance indicated greater stability of upstream gene transcription.

The relative abundance of transcription was divided into three intervals: "1–4", "4–7", and ">7" (Fig. 2D). In the group with higher relative abundance of transcription, more terminator variants were from strong terminator bins (bin 2 and bin 3). For instance, 70 % of terminator variants in the group with relative abundance of transcript >7 belonged to bin 3, and less than 20 % belonged to bin 1. These results confirmed that the positive correlation between the abundance of upstream gene transcripts with the terminator strength, which was defined by the downstream gene expression. Since the gene background were the same among all terminator variants, including promoter, 5′UTR, CDS, we argue that the terminator's hairpin structure effectively inhibits the hydrolyzation of RNase hydrolase enzyme, thereby slowing down mRNA degradation [11,41].

### 3.2 Sequence characteristics of terminators ascribed to higher upstream mRNA stability

The terminator variants in this terminator mutation library were predicted to adopt a stem-loop structure with 13-15 nt stem and 6 nt loop. The base of the hairpin is located at positions M12-M13 and M46-M47, while positions M56-M58 are situated downstream of the U-tract. A strong correlation between terminating efficiency and the nucleotide composition at the base of the hairpin and downstream of the U-tract was reported [31,32]. To further explore the structural features of the terminator that affect the stability of the upstream mRNA, we compared base differences at the hairpin base and U-tract downstream positions of terminators with different transcript abundance.

**Fig. 3:**
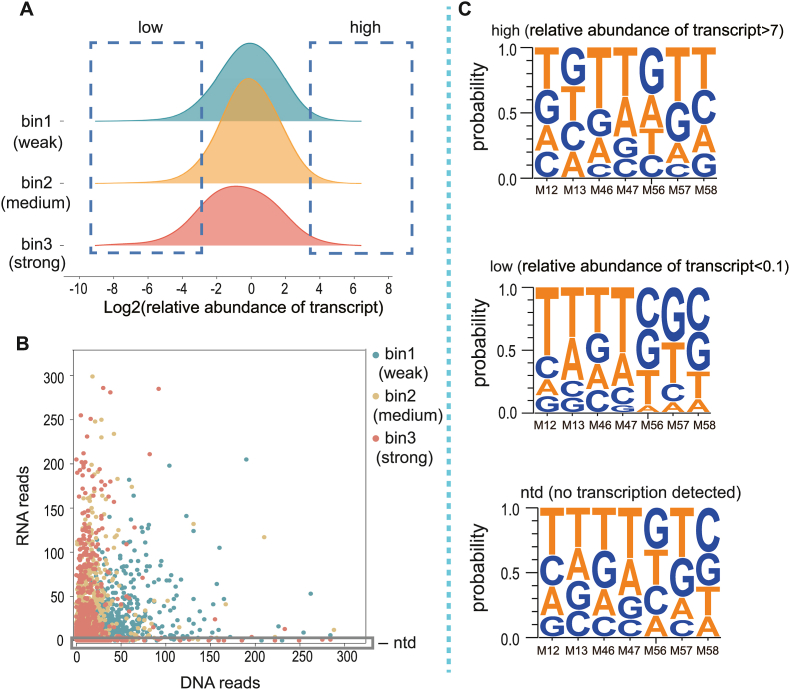
The relative abundance of mRNA compared to DNA abundance in three bins, as well as the sequence features of terminators belonging to groups with high RNA abundance, low RNA abundance, and no detected transcripts. A. The classification of mRNA abundance groups was performed based on a relative transcript abundance cutoff of >7 (high) and <0.1 (low); B. The terminators with upstream mRNA reads = 0 were classified as the "ntd" group. C. Nucleotide compositions of each site of terminator variants in three groups.

Three groups of terminators with extreme relative mRNA abundance were chosen for structure-activity analysis. Terminators exhibiting a strong ability to maintain the upstream mRNA stability were classified as "high mRNA abundance group" (relative abundance of transcript >7), while those with a weak ability were categorized as "low mRNA abundance group" (relative abundance of transcript <0.1) (Fig. 3A). Additionally, certain terminator variants, which failed to generate any detectable RNA reads, suggested complete degradation by RNase. These particular terminator variants were assigned to the "ntd group" (no transcription detected) (Fig. 3B). The sequence differences between groups were compared (Fig. 3C). The result revealed that the frequency of GC in the "low" group was much lower than that observed in the "high" group. The frequency of AT in the "high" group exceeded 0.5 at the downstream of U-tract (M56-M58), whereas it was only 0.4 in the "low" group. The terminators in the "ntd" group and "low" group showed very similar sequence features.

**Fig. 4:**
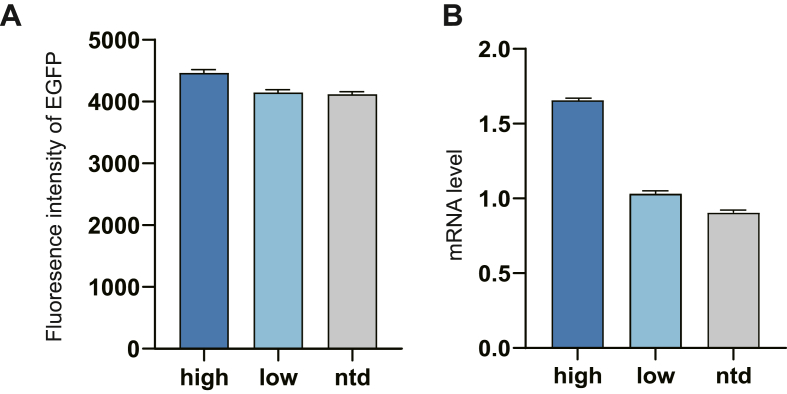
Fluoresence intensity of EGFP and upstream mRNA level under the regulation of most conserved terminator sequences obtained in the high, low and ntd groups. Three biological replicates were measured.

In order to verify the effect of terminators on upstream gene transcription in the high, low and ntd groups, we designed the three most representative terminators according to the results of Fig. 3C, and examined the fluoresence intensity of EGFP and upstream mRNA level under the regulation of these three terminators, and the results are shown in Fig. 4A and B. From the high group to the low group to the ntd group, the fluoresence intensity of EGFP and mRNA level under the regulation of the terminators gradually decreased, which was consistent with the conclusions obtained earlier. In general, terminators with the feature of high GC content at the base of the hairpin and high AT content at the downstream of the U-tract are more conducive to the maintenance of upstream mRNA stability. Interestingly, Chen et al. showed that terminators with GC rich at the base of the hairpin and AT-rich at the downstream of the U-tract had higher termination efficiencies, which further validated our conclusion that strong terminators are more favorable for maintaining upstream mRNA stability.

### 3.3 Secondary structure of the terminators associated with upstream mRNA stability

Endogenous terminators terminate transcription by folding RNA into secondary structures, and variations in these structures significantly impact the efficiency of termination. To investigate whether the secondary structure of a terminator also influences its function in maintaining upstream gene mRNA stability, minimum free energy (ΔG), hairpin stem length, and base pairing at the base of the hairpin were predicted, and their correlation with upstream gene mRNA abundance were analyzed.

**Fig. 5:**
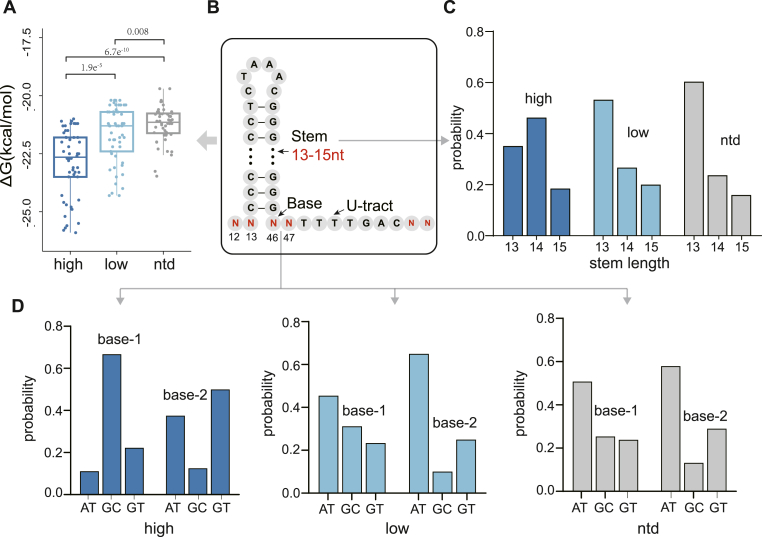
Secondary structure of the terminators in the "high", "low" and "ntd" groups. A. Free energy *ΔG* of the terminator in the "high", "low" and "ntd" groups. B. The sequence and structure terminator used in this study. C. The stem length of the terminator in the "high", "low" and "ntd" groups. D. The predicted base-pairing of the hairpin base region of the "high", "low" and "ntd" groups.

The results showed that the terminators in the high mRNA abundance group exhibited minimal free energy ΔG much lower than those in the "low" and "ntd" groups (−24 to −19.5 kcal/mol). The minimum free energy ΔG of the terminators decreases steadily from "ntd" to "low" to "high", indicating that terminators with lower free energy ΔG are more conducive to maintaining upstream mRNA stability (Fig. 5A).

The "high" group comprised the highest proportion of terminators with 14 nt stem, reaching 50 % (Fig. 5C). In comparison, the "low" and "ntd" groups comprised the highest proportion of terminators with 13 nt stem, surpassing 50 %. Overall, terminators in the "high" group generally possessed longer stem lengths, suggesting that terminators with longer stems better protect the stability of upstream gene mRNAs.

There are 16 different combinations of nucleotides at the base of the hairpin, but only three combinations, G + C, G + T and A + T, can be linked by hydrogen bonds to form pairs. As for the base of the hairpin in the three groups, the probability of G + C and G + T pairing is higher than the probability of A + T pairing in the "high" group. In contrast, the probability of A + T pairing is greater than the probability of G + C and G + T pairing in the "low" and "ntd" groups (Fig. 5D). Such results suggest that terminators with G + C and G + T base-pairing at the base of the hairpin are more advantageous for maintaining upstream mRNA stability.

In summary, terminators exhibiting lower free energy ΔG, longer stem length, G + C or G + T base-pairing at the base of the hairpin are positively correlated with upstream mRNA abundance. It is important to note that these characteristics are primarily associated with termination efficiency [[31], [32], [33], [34]], indicating a close relationship between terminating efficiency and upstream mRNA stability.

### 3.4 The validation of terminator structural characteristics that enhance upstream mRNA stability

**Fig. 6:**
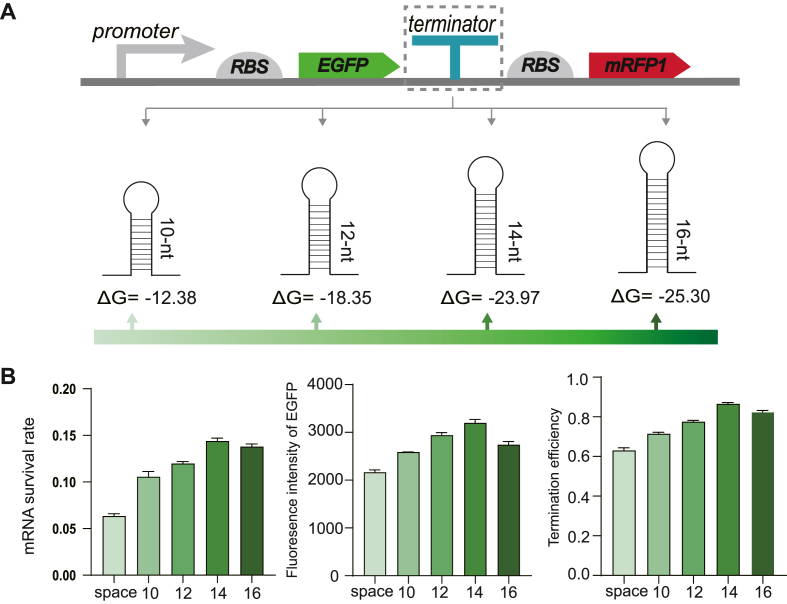
mRNA survival rate and fluorescence intensity of EGFP under terminator regulation with different stem lengths and free energies ΔG. A. Free energy of terminators with 10 nt, 12 nt, 14 nt, and 16 nt stem; B. mRNA survival rate, fluorescence intensity of EGFP and termination efficiency, where “space” is the control group without terminator. Three biological replicates were measured.

The above results obtained by FlowSeq and high throughput sequencing revealed that longer stem length and lower free energy (ΔG) of the terminator benefit the upstream mRNA stability. To validate this structure-activity correlation in different genetic backgrounds, we modified the dual fluorescent probe plasmid PTK by replacing the expression system from *E. coli* BL21 (DE3) to *E. coli* JM109 and substituting the original T7 promoter with tac promoter. The mRNA survival rate and fluorescence intensity of upstream reporter gene (EGFP) were quantified under terminators with varying stem lengths and free energies. The mRNA survival rate can be assessed by the addition of rifampicin at a specific time point to inhibit further RNA transcription, and by quantifying the initial and final levels of transcript abundance via qPCR, mRNA survival rate can be determined. The minimum free energy (ΔG) predicted continuously decreased as the stem length increased from 10 to 14 nt (Fig. 6A). Additionally, a control group was constructed that contains space sequence instead of terminator. this group lacks the activity to terminate transcription.

It was observed that the mRNA survival rate regulated by terminators (with varied stem lengths) was 18 %–40 % higher compared to that of the space group (Fig. 6). mRNA stabilization positively correlated with terminator stem length in the range of 10 nt to 14 nt. However, the mRNA survival rate decreased instead when the stem length exceeded 14 nt. Similar trends were observed in fluorescence intensity results. Therefore, terminators with relatively longer stem (14 nt) enhanced protective effect on mRNA stability, and further enhanced protein expression.

RNase III recognizes the stem-loop structure of mRNA for directional cleavage, and at the same time attracts RNase E or RNase G for further cleavage, resulting in total degradation of mRNA [11,40,48]. The terminator forms a secondary structure through self-folding of its RNA sequence, thereby obstructing the binding of 3′-mRNA to RNase E, and consequently safeguarding mRNA from degradation. Therefore, from this perspective, the greater stability exhibited by the secondary structure of the terminator enhanced protective effect against hydrolases. However, excessive stem length in terminator facilitates recruitment of RNase III for targeted cleavage, resulting in "double-edged sword effect" of the terminator stem. The validation results here. Overall, confirmed the aforementioned hydrolysis resistant effect of terminator, and specifically, terminators with a stem length of 14 nt demonstrated optimal efficacy in preserving the mRNA stability of upstream gene.

### 3.5 Using optimal terminator to enhance mRNA level and subsequent fermentation yields in diverse expression systems

Finally, we illustrated the application of optimal terminators characterized above to modulate the gene expression, especially the mRNA level, in varied gene backgrounds other than the probe plasmid PTK-EGFP-mRFP1 in the *Escherichia coli* system, three expression systems in *E*. *coli* or *B. subtilis* with NMN, SAM and 25(OH)VD3 as the final fermentation product were tested.

**Fig. 7:**
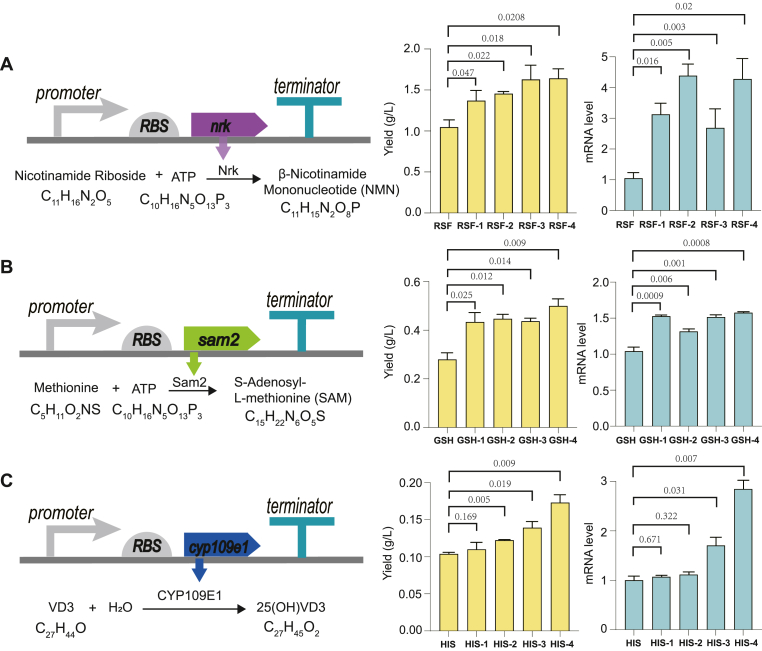
The fermentation production and mRNA level observed in parental strain and the mutant strain regulated by optimal terminators. A. The fermentation product (NMN) and *nrk* transcripts were compared under the regulation of varied terminators. B. The fermentation product (SAM) and *sam2* transcripts were compared under the regulation of varied terminators. C. The fermentation product (25(OH)VD3) and *cyp109e1* transcripts were compared under the regulation of varied terminators. Three biological replicates were measured.

First, we employed optimal terminators to regulate the expression of nicotinamide ribose kinase (Nrk) in *E*. *coli* RSF, which constitutes a crucial step for NMN synthesis. Four terminators (h-1 to h-4) from the "high" group, demonstrating superior efficacy in maintaining mRNA stability of upstream gene, were applied to regulate Nrk expression. Specially, the sequence characterization and secondary structure characterization of h-1 to h-4 were shown in Table S3. The terminators were integrated downstream of *nrk* in the plasmid of strain RSF (promoter: T7 promoter; gene: *nrk*; terminator: T7 terminator), resulting in the generation of four mutant strains designated as RSF-1 to RSF-4. As illustrated in Fig. 7A, a significant upregulation was observed in the mRNA levels of the upstream gene in recombinant strains RSF-1 to RSF-4 compared to the parental strain RSF, with an impressive four-fold increase observed specifically in strain RSF-4. Moreover, while the parental strain produced 1.0 g/L NMN, insertion of terminators led to varying degrees of enhanced production. Notably, strain RSF-4 harboring terminator h-4 exhibited the highest NMN production (1.64 g/L), representing a remarkable 64 % improvement over its parental strain.

The S-adenosylmethionine synthetase (Sam2) derived from *S. cerevisiae* was expressed in *E. coli* GSH, which served as the parental strain (promoter: T7 promoter; gene: *sam2*; terminator: T7 terminator). Mutant strains GSH-1 to GSH-4 were constructed by incorporating terminators h-1 to h-4 into the plasmid of the GSH. Fig. 7 B illustrated mRNA level of Sam2 was significant upregulated in the range of 30 %–55 % for all recombinant strains (GSH-1 to GSH-4) when compared with their parental strain. Moreover, the SAM production by GSH was 0.28 g/L, while all recombinant strains (GSH-1 to GSH-4) exhibited higher yields, representing a remarkable increase of 40 %–60 % compared to that of parental strain.

Finally, CYP109E1 was expressed in *B. subtilis* HIS, and used as the parental strain (promoter: Hpa II promoter; gene: *sam2*; terminator: fd terminator). Four mutant strains (HIS-1 to HIS-4) were generated by incorporating terminators h-1 to h-4 into the plasmid of HIS, which effectively protected the mRNA stability of upstream genes. The production of 25(OH)VD3 and mRNA levels of upstream genes in both HIS and HIS-1 to HIS-4 were examined (Fig. 7C). The upstream gene mRNA levels of the mutant strains were improved compared with that of the parental strain HIS, with HIS-4 harboring terminator h-4 exhibited the highest level of mRNA, which is almost 3-fold of that in parental strain. The parental strain HIS exhibited a fermentation product yield of 0.10 g/L for 25(OH)VD3. Compared to this, the mutant strains enhanced 25(OH)VD3 production to varied extend, with a maximum yield of 0.17 g/L achieved by HIS-4, representing a remarkable improvement of 70 % over that obtained from HIS.

The above results verified that the optimal terminators chosen from the "high" group (h-1 to h-4) improved the fermentation product yield and upstream mRNA level in three different expression systems, and confirmed the connection between the structural characteristics of terminators and upstream mRNA stability, as well as the overall gene expression.

However, upon closer examination of fermentation production and upstream mRNA levels, the correlation between these two phenotypical changes in the recombinant strain harboring h1-h4 terminators is not entirely consistent. This inconsistency is particularly evident in the NMN production system, where the highest mRNA level and NMN production were observed in strains harboring h4 and h2 terminators respectively. Overall, these results in *E*. *coli* and *B*. *subtilis* demonstrated that terminator h4 exhibited superior efficacy in enhancing upstream gene expression across multiple gene backgrounds, and further improved fermentation production. It shared the same high relative abundance of transcript and low free energy ΔG as h-1 to h-3, but with a stem length of 14 nt, which further validated that a terminator with a stem length of 14 nt can better protect the upstream gene mRNA stability.

## 4 Conclusions

Comprehending the function of terminators in maintaining mRNA stability is crucial for the proactive discovery of gene expression regulators. In this study, by FlowSeq technology, terminator variants characterized by their regulation effect on mRNA abundance and protein expression. Our initial findings indicate that strong terminators are more advantageous in maintaining mRNA stability. The analysis of the correlation between the sequence characteristics of terminators and upstream mRNA stability showed that terminators with GC-rich at the base of the hairpin and AT-rich at the downstream of the U-tract are more conducive to the maintenance of upstream mRNA stability. Moreover, terminators with lower free energy and longer stem lengths are more effective at preserving upstream mRNA stability, and terminators with 14 nt stem had the best protective effect on upstream mRNA stability. The optimal terminators selected in the terminator library were further tested in different expression systems across species, and the features of terminators ascribed to enhancing upstream gene stability were verified. The production NMN and SAM were increased by 40 %–70 %, the production of 25(OH)VD3 were increased by 10 %–70 %, and the mRNA levels of the upstream genes (*nrk*, *sam2*, and *cyp109e1*) were also increased to varied levels. Our study regarding the characteristics of terminators that contribute to the upstream mRNA stability provided valuable principles for the rational design of terminators beneficial for upstream gene expression.

## CRediT authorship contribution statement

**Jia-Wei Ren:** Writing – original draft, Methodology, Data curation. **Jin-Peng Zhang:** Software, Data curation. **Zi-Lun Mei:** Data curation. **Jia-Yi Shao:** Data curation. **Guo-Qiang Xu:** Writing – review & editing. **Hui Li:** Writing – original draft, Resources. **Jin-Song Gong:** Resources. **Xiao-Mei Zhang:** Writing – review & editing. **Jin-Song Shi:** Writing – review & editing. **Xiao-juan Zhang:** Supervision, Funding acquisition, Conceptualization. **Zheng-hong Xu:** Supervision, Conceptualization.

## Declaration of competing interest

The authors declare that they have no known competing financial interests or personal relationships that could have appeared to influence the work reported in this paper.
