# Supplementary material for: Regulatory significance of terminator: A systematic approach for dissecting terminator-mediated enhancement of upstream mRNA stability
Source: Synth Syst Biotechnol. 2024 Nov 28;10(1):326–35. doi: 10.1016/j.synbio.2024.11.006 (PMC11696848; doi:10.1016/j.synbio.2024.11.006)
Supplement: Multimedia component 1 [file mmc1.docx]

**Figure S1**


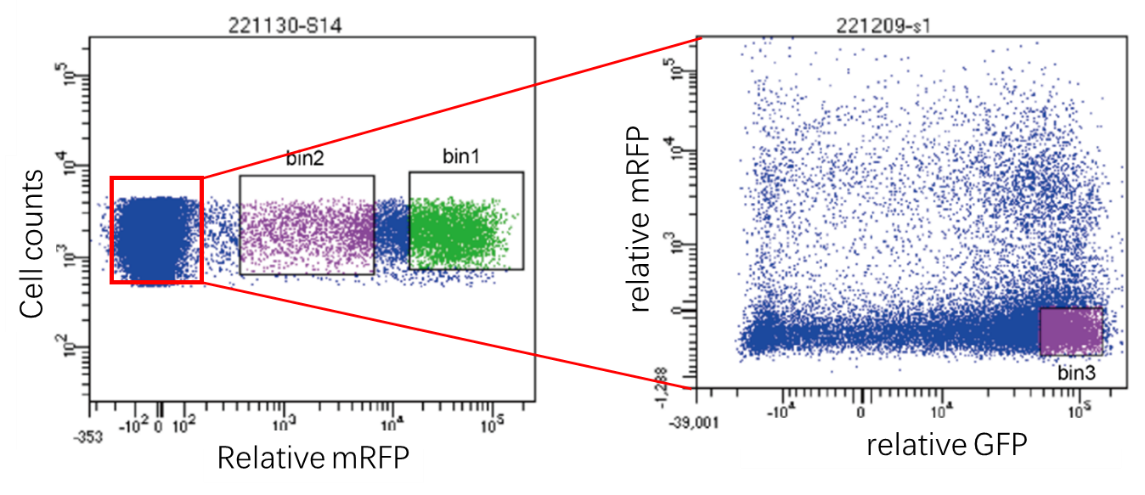


Supplementary Figure S1.Results of grouping mutant libraries by downstream red fluorescent expression level by flow cytometry.

**Supplementary Note 1: Sequences that fulfill the criteria of the "high" group**

| **Sequence** | Relative abundance of transcript |
| --- | --- |
| AAAACTAGCATAGCCCCTTGGGGCCTCTAAACGGGTCTTGAGGGGAATTTTGACGGGC | 41.1496 |
| AAAACTAGCATTTCCCCTTGGGGCCTCTAAACGGGTCTTGAGGGGTGTTTTGACGATT | 31.25205 |
| AAAACTAGCATAGCCCCTTGGGGCCTCTAAACGGGTCTTGAGGGGGGTTTTGACGGCT | 30.55446 |
| AAAACTAGCATCCCCCCTTGGGGCCTCTAAACGGGTCTTGAGGGGTGTTTTGACGTCC | 22.04386 |
| AAAACTAGCATTGCCCCTTGGGGCCTCTAAACGGGTCTTGAGGGGAATTTTGACGGTA | 20.21515 |
| AAAACTAGCATATCCCCTTGGGGCCTCTAAACGGGTCTTGAGGGGTGTTTTGACGATC | 18.97495 |
| AAAACTAGCATTCCCCCTTGGGGCCTCTAAACGGGTCTTGAGGGGGCTTTTGACGGGC | 17.95179 |
| AAAACTAGCATAACCCCTTGGGGCCTCTAAACGGGTCTTGAGGGGAATTTTGACGCTC | 17.27202 |
| AAAACTAGCATGGCCCCTTGGGGCCTCTAAACGGGTCTTGAGGGGTTTTTTGACGGTC | 16.45323 |
| AAAACTAGCATCACCCCTTGGGGCCTCTAAACGGGTCTTGAGGGGTGTTTTGACGTTC | 14.1382 |
| AAAACTAGCATGACCCCTTGGGGCCTCTAAACGGGTCTTGAGGGGTTTTTTGACGGTT | 13.95181 |
| AAAACTAGCATTGCCCCTTGGGGCCTCTAAACGGGTCTTGAGGGGTGTTTTGACGGCG | 13.63291 |
| AAAACTAGCATTCCCCCTTGGGGCCTCTAAACGGGTCTTGAGGGGTTTTTTGACGGTT | 13.39374 |
| AAAACTAGCATAACCCCTTGGGGCCTCTAAACGGGTCTTGAGGGGTTTTTTGACGGTT | 12.83566 |
| AAAACTAGCATCACCCCTTGGGGCCTCTAAACGGGTCTTGAGGGGTTTTTTGACGGTT | 12.55663 |
| AAAACTAGCATGCCCCCTTGGGGCCTCTAAACGGGTCTTGAGGGGGGTTTTGACGTGA | 12.52595 |
| AAAACTAGCATACCCCCTTGGGGCCTCTAAACGGGTCTTGAGGGGATTTTTGACGAAT | 11.9288 |
| AAAACTAGCATTTCCCCTTGGGGCCTCTAAACGGGTCTTGAGGGGAATTTTGACGCTC | 11.53379 |
| AAAACTAGCATTCCCCCTTGGGGCCTCTAAACGGGTCTTGAGGGGTTTTTTGACGAAC | 11.06215 |
| AAAACTAGCATCTCCCCTTGGGGCCTCTAAACGGGTCTTGAGGGGTTTTTTGACGGTT | 11.04983 |
| AAAACTAGCATATCCCCTTGGGGCCTCTAAACGGGTCTTGAGGGGTTTTTTGACGGGT | 10.63023 |
| AAAACTAGCATTCCCCCTTGGGGCCTCTAAACGGGTCTTGAGGGGTATTTTGACGTGG | 9.939261 |
| AAAACTAGCATTGCCCCTTGGGGCCTCTAAACGGGTCTTGAGGGGAGTTTTGACGCTA | 9.859534 |
| AAAACTAGCATGTCCCCTTGGGGCCTCTAAACGGGTCTTGAGGGGCTTTTTGACGGGA | 9.580491 |
| AAAACTAGCATTCCCCCTTGGGGCCTCTAAACGGGTCTTGAGGGGTGTTTTGACGATC | 9.566957 |
| AAAACTAGCATGCCCCCTTGGGGCCTCTAAACGGGTCTTGAGGGGTATTTTGACGCTT | 9.25494 |
| AAAACTAGCATTACCCCTTGGGGCCTCTAAACGGGTCTTGAGGGGTTTTTTGACGCCT | 9.141994 |
| AAAACTAGCATCGCCCCTTGGGGCCTCTAAACGGGTCTTGAGGGGATTTTTGACGGTT | 9.115419 |
| AAAACTAGCATAGCCCCTTGGGGCCTCTAAACGGGTCTTGAGGGGGCTTTTGACGGTA | 9.053409 |
| AAAACTAGCATCTCCCCTTGGGGCCTCTAAACGGGTCTTGAGGGGGTTTTTGACGAGG | 9.032158 |
| AAAACTAGCATGGCCCCTTGGGGCCTCTAAACGGGTCTTGAGGGGTGTTTTGACGTGC | 8.92939 |
| AAAACTAGCATGGCCCCTTGGGGCCTCTAAACGGGTCTTGAGGGGATTTTTGACGGGG | 8.624692 |
| AAAACTAGCATTTCCCCTTGGGGCCTCTAAACGGGTCTTGAGGGGTATTTTGACGGAG | 8.557332 |
| AAAACTAGCATTCCCCCTTGGGGCCTCTAAACGGGTCTTGAGGGGCTTTTTGACGGAC | 8.48292 |
| AAAACTAGCATGCCCCCTTGGGGCCTCTAAACGGGTCTTGAGGGGGGTTTTGACGGGT | 8.464317 |
| AAAACTAGCATAGCCCCTTGGGGCCTCTAAACGGGTCTTGAGGGGTATTTTGACGATG | 8.298292 |
| AAAACTAGCATGTCCCCTTGGGGCCTCTAAACGGGTCTTGAGGGGATTTTTGACGCTC | 8.185274 |
| AAAACTAGCATTGCCCCTTGGGGCCTCTAAACGGGTCTTGAGGGGTCTTTTGACGTCA | 8.185274 |
| AAAACTAGCATAGCCCCTTGGGGCCTCTAAACGGGTCTTGAGGGGTATTTTGACGCTG | 7.922946 |
| AAAACTAGCATGGCCCCTTGGGGCCTCTAAACGGGTCTTGAGGGGCATTTTGACGAAC | 7.896536 |
| AAAACTAGCATTGCCCCTTGGGGCCTCTAAACGGGTCTTGAGGGGTATTTTGACGTTA | 7.843716 |
| AAAACTAGCATCTCCCCTTGGGGCCTCTAAACGGGTCTTGAGGGGGTTTTTGACGTAC | 7.813216 |
| AAAACTAGCATTTCCCCTTGGGGCCTCTAAACGGGTCTTGAGGGGAATTTTGACGTGG | 7.813012 |
| AAAACTAGCATACCCCCTTGGGGCCTCTAAACGGGTCTTGAGGGGTTTTTTGACGGTT | 7.813012 |
| AAAACTAGCATTACCCCTTGGGGCCTCTAAACGGGTCTTGAGGGGGCTTTTGACGTAA | 7.738804 |
| AAAACTAGCATTTCCCCTTGGGGCCTCTAAACGGGTCTTGAGGGGGCTTTTGACGGTA | 7.724872 |
| AAAACTAGCATTTCCCCTTGGGGCCTCTAAACGGGTCTTGAGGGGATTTTTGACGTCA | 7.653762 |
| AAAACTAGCATCCCCCCTTGGGGCCTCTAAACGGGTCTTGAGGGGGATTTTGACGCTT | 7.51557 |
| AAAACTAGCATAGCCCCTTGGGGCCTCTAAACGGGTCTTGAGGGGAATTTTGACGCGG | 7.447569 |
| AAAACTAGCATCTCCCCTTGGGGCCTCTAAACGGGTCTTGAGGGGAATTTTGACGGAT | 7.441158 |
| AAAACTAGCATGGCCCCTTGGGGCCTCTAAACGGGTCTTGAGGGGGCTTTTGACGCCG | 7.28911 |
| AAAACTAGCATGCCCCCTTGGGGCCTCTAAACGGGTCTTGAGGGGTTTTTTGACGGTT | 7.25494 |
| AAAACTAGCATCTCCCCTTGGGGCCTCTAAACGGGTCTTGAGGGGCATTTTGACGTGT | 7.25494 |
| AAAACTAGCATTGCCCCTTGGGGCCTCTAAACGGGTCTTGAGGGGGTTTTTGACGATA | 7.162115 |
| AAAACTAGCATTACCCCTTGGGGCCTCTAAACGGGTCTTGAGGGGCTTTTTGACGACC | 7.130651 |
| AAAACTAGCATCACCCCTTGGGGCCTCTAAACGGGTCTTGAGGGGCATTTTGACGTGT | 7.115422 |

**Supplementary Note 2: Plasmid Information**

**PTK**

AGAAAAATAAACAAAAGAGTTTGTAGAAACGCAAAAAGGCCATCCGTCAGGATGGCCTTCTGCTTAATTTGATGCCTGGCAGTTTATGGCGGGCGTCCTGCCCGCCACCCTCCGGGCCGTTGCTTCGCAACGTTCAAATCCGCTCCCGGCGGATTTGTCCTACTCAGGAGAGCGTTCACCGACAAACAACAGATAAAACGAAAGGCCCAGTCTTTCGACTGAGCCTTTCGTTTTATTTGATGCCTGGCAGTTCCCTACTCTCGCATTGTGCGCGGAACCCCTATTTGTTTATTTTTCTAAATACATTCAAATATGTATCCGCTCATGAATTAATTCTTAGAAAAACTCATCGAGCATCAAATGAAACTGCAATTTATTCATATCAGGATTATCAATACCATATTTTTGAAAAAGCCGTTTCTGTAATGAAGGAGAAAACTCACCGAGGCAGTTCCATAGGATGGCAAGATCCTGGTATCGGTCTGCGATTCCGACTCGTCCAACATCAATACAACCTATTAATTTCCCCTCGTCAAAAATAAGGTTATCAAGTGAGAAATCACCATGAGTGACGACTGAATCCGGTGAGAATGGCAAAAGTTTATGCATTTCTTTCCAGACTTGTTCAACAGGCCAGCCATTACGCTCGTCATCAAAATCACTCGCATCAACCAAACCGTTATTCATTCGTGATTGCGCCTGAGCGAGACGAAATACGCGATCGCTGTTAAAAGGACAATTACAAACAGGAATCGAATGCAACCGGCGCAGGAACACTGCCAGCGCATCAACAATATTTTCACCTGAATCAGGATATTCTTCTAATACCTGGAATGCTGTTTTCCCGGGGATCGCAGTGGTGAGTAACCATGCATCATCAGGAGTACGGATAAAATGCTTGATGGTCGGAAGAGGCATAAATTCCGTCAGCCAGTTTAGTCTGACCATCTCATCTGTAACATCATTGGCAACGCTACCTTTGCCATGTTTCAGAAACAACTCTGGCGCATCGGGCTTCCCATACAATCGATAGATTGTCGCACCTGATTGCCCGACATTATCGCGAGCCCATTTATACCCATATAAATCAGCATCCATGTTGGAATTTAATCGCGGCCTAGAGCAAGACGTTTCCCGTTGAATATGGCTCATAACACCCCTTGTATTACTGTTTATGTAAGCAGACAGTTTTATTGTTCATGACCAAAATCCCTTAACGTGAGTTTTCGTTCCACTGAGCGTCAGACCCCGTAGAAAAGATCAAAGGATCTTCTTGAGATCCTTTTTTTCTGCGCGTAATCTGCTGCTTGCAAACAAAAAAACCACCGCTACCAGCGGTGGTTTGTTTGCCGGATCAAGAGCTACCAACTCTTTTTCCGAAGGTAACTGGCTTCAGCAGAGCGCAGATACCAAATACTGTTCTTCTAGTGTAGCCGTAGTTAGGCCACCACTTCAAGAACTCTGTAGCACCGCCTACATACCTCGCTCTGCTAATCCTGTTACCAGTGGCTGCTGCCAGTGGCGATAAGTCGTGTCTTACCGGGTTGGACTCAAGACGATAGTTACCGGATAAGGCGCAGCGGTCGGGCTGAACGGGGGGTTCGTGCACACAGCCCAGCTTGGAGCGAACGACCTACACCGAACTGAGATACCTACAGCGTGAGCTATGAGAAAGCGCCACGCTTCCCGAAGGGAGAAAGGCGGACAGGTATCCGGTAAGCGGCAGGGTCGGAACAGGAGAGCGCACGAGGGAGCTTCCAGGGGGAAACGCCTGGTATCTTTATAGTCCTGTCGGGTTTCGCCACCTCTGACTTGAGCGTCGATTTTTGTGATGCTCGTCAGGGGGGCGGAGCCTATGGAAAAACGCCAGCAACGCGGCCTTTTTACGGTTCCTGGCCTTTTGCTGGCCTTTTGCTCACATGTTCTTTCCTGCGTTATCCCCTGATTCTGTGGATAACCGTATTACCGCCTTTGAGTGAGCTGATACCGCTCGCCGCAGCCGAACGACCGAGCGCAGCGAGTCAGTGAGCGAGGAAGCCCTAGCCGGATATAGTTCCTCCTTTCAGCAAAAAACCCCTCAAGACCCGTTTAGAGGCCCCAAGGGGTTATGCTAGTTATTGCTCAGCGGTGGCAGCAGCCAACTCAGCTTCCTTTCGGGCTTTGTTAGCAGCCGGATCTCAGTGGTGGTGGTGGTGGTGGACACCATCGAATGGTGCAAAACCTTTCGCGGTATGGCATGATAGCGCCCGGAAGAGAGTCAATTCAGGGTGGTGAATGTGAAACCAGTAACGTTATACGATGTCGCAGAGTATGCCGGTGTCTCTTATCAGACCGTTTCCCGCGTGGTGAACCAGGCCAGCCACGTTTCTGCGAAAACGCGGGAAAAAGTGGAAGCGGCGATGGCGGAGCTGAATTACATTCCCAACCGCGTGGCACAACAACTGGCGGGCAAACAGTCGTTGCTGATTGGCGTTGCCACCTCCAGTCTGGCCCTGCACGCGCCGTCGCAAATTGTCGCGGCGATTAAATCTCGCGCCGATCAACTGGGTGCCAGCGTGGTGGTGTCGATGGTAGAACGAAGCGGCGTCGAAGCCTGTAAAGCGGCGGTGCACAATCTTCTCGCGCAACGCGTCAGTGGGCTGATCATTAACTATCCGCTGGATGACCAGGATGCCATTGCTGTGGAAGCTGCCTGCACTAATGTTCCGGCGTTATTTCTTGATGTCTCTGACCAGACACCCATCAACAGTATTATTTTCTCCCATGAAGACGGTACGCGACTGGGCGTGGAGCATCTGGTCGCATTGGGTCACCAGCAAATCGCGCTGTTAGCGGGCCCATTAAGTTCTGTCTCGGCGCGTCTGCGTCTGGCTGGCTGGCATAAATATCTCACTCGCAATCAAATTCAGCCGATAGCGGAACGGGAAGGCGACTGGAGTGCCATGTCCGGTTTTCAACAAACCATGCAAATGCTGAATGAGGGCATCGTTCCCACTGCGATGCTGGTTGCCAACGATCAGATGGCGCTGGGCGCAATGCGCGCCATTACCGAGTCCGGGCTGCGCGTTGGTGCGGATATCTCGGTAGTGGGATACGACGATACCGAAGACAGCTCATGTTATATCCCGCCGTTAACCACCATCAAACAGGATTTTCGCCTGCTGGGGCAAACCAGCGTGGACCGCTTGCTGCAACTCTCTCAGGGCCAGGCGGTGAAGGGCAATCAGCTGTTGCCCGTCTCACTGGTGAAAAGAAAAACCACCCTGGCGCCCAATACGCAAACCGCCTCTCCCCGCGCGTTGGCCGATTCATTAATGCAGCTGGCACGACAGGTTTCCCGACTGGAAAGCGGGCAGTGACTGTGCAGGTCGTAAATCACTGCATAATTCGTGTCGCTCAAGGCGCACTCCCGTTCTGGATAATGTTTTTTGCGCCGACATCATAACGGTTCTGGCAAATATTCTGAAATGAATTGGTTTTCCCAGTCACGACCCTAGGGAGCTGTAATACGACTCACTATATGTGGAATTGTGAGCGGATAACAATTAACTAACT*AAGAAAGGACATGAACG*ATGGTGAGCAAGGGCGAGGAGCTGTTCACCGGGGTGGTGCCCATCCTGGTCGAGCTGGACGGCGACGTAAACGGCCACAAGTTCAGCGTGTCCGGCGAGGGCGAGGGCGATGCCACCTACGGCAAGCTGACCCTGAAGTTCATCTGCACCACCGGCAAGCTGCCCGTGCCCTGGCCCACCCTCGTGACCACCCTGACCTACGGCGTGCAGTGCTTCAGCCGCTACCCCGACCACATGAAGCAGCACGACTTCTTCAAGTCCGCCATGCCCGAAGGCTACGTCCAGGAGCGCACCATCTTCTTCAAGGACGACGGCAACTACAAGACCCGCGCCGAGGTGAAGTTCGAGGGCGACACCCTGGTGAACCGCATCGAGCTGAAGGGCATCGACTTCAAGGAGGACGGCAACATCCTGGGGCACAAGCTGGAGTACAACTACAACAGCCACAACGTCTATATCATGGCCGACAAGCAGAAGAACGGCATCAAGGTGAACTTCAAGATCCGCCACAACATCGAGGACGGCAGCGTGCAGCTCGCCGACCACTACCAGCAGAACACCCCCATCGGCGACGGCCCCGTGCTGCTGCCCGACAACCACTACCTGAGCACCCAGTCCGCCCTGAGCAAAGACCCCAACGAGAAGCGCGATCACATGGTCCTGCTGGAGTTCGTGACCGCCGCCGGGATCACTCTCGGCATGGACGAGCTGTACAAGTAAGCTAGCGAATTCGTCGACGGAACTCAAGCTTTAACTAACT*AAGAAAGGACATGAACG*ATGGCCTCCTCCGAGGACGTCATCAAGGAGTTCATGCGCTTCAAGGTGCGCATGGAGGGCTCCGTGAACGGCCACGAGTTCGAGATCGAGGGCGAGGGCGAGGGCCGCCCCTACGAGGGCACCCAGACCGCCAAGCTGAAGGTGACCAAGGGCGGCCCCCTGCCCTTCGCCTGGGACATCCTGTCCCCTCAGTTCCAGTACGGCTCCAAGGCCTACGTGAAGCACCCCGCCGACATCCCCGACTACTTGAAGCTGTCCTTCCCCGAGGGCTTCAAGTGGGAGCGCGTGATGAACTTCGAGGACGGCGGCGTGGTGACCGTGACCCAGGACTCCTCCCTGCAGGACGGCGAGTTCATCTACAAGGTGAAGCTGCGCGGCACCAACTTCCCCTCCGACGGCCCCGTAATGCAGAAGAAGACCATGGGCTGGGAGGCCTCCACCGAGCGGATGTACCCCGAGGACGGCGCCCTGAAGGGCGAGATCAAGATGAGGCTGAAGCTGAAGGACGGCGGCCACTACGACGCCGAGGTCAAGACCACCTACATGGCCAAGAAGCCCGTGCAGCTGCCCGGCGCCTACAAGACCGACATCAAGCTGGACATCACCTCCCACAACGAGGACTACACCATCGTGGAACAGTACGAGCGCGCCGAGGGCCGCCACTCCACCGGCGCCTAA

Gray: T7 operator

Green: EGFP gene

Red: mRFP1 gene

**RSF**

GACGCTCTCCCTTATGCGACTCCTGCATTAGGAAATTAATACGACTCACTATAGGGGAATTGTGAGCGGATAACAATTCCCCTGTAGAAATAATTTTGTTTAACTTTAATAAGGAGATATACCATGGGCAGCAGCCATCACCATCATCACCACAGCCAGGATCCGATGACGACAACTAAAGTTAAATTGATCGCAATTTCAGGCTGCTCTTCCTCGGGCAAAACGACACTGGCCAAATTTCTGGCGAACGCGATCCCAGGTTGTATTCTTATTCACGAAGATGACTTCTATAAACCTGATTCGGAGATCCCGATCAACGAAAAATATGGAGTCGCGGACTGGGATTGCCCGGAGGCCCTGGACTTGGACGCGTTCAAACGTGAGTTGGACTTGATTAAAACCACAGGTTCGATTAAAACGAAACTTATCCACACGTCTACTAGCGCAGCTTAATTAACCTAGGCTGCTGCCACCGCTGAGCAATAACTAGCATAACCCCTTGGGGCCTCTAAACGGGTCTTGAGGGGTTTTTTGCTGAAACCTCAGGCATTTGAGAAGCACACGGTCACACTGCTTCCGGTAGTCAATAAACCGGTAAACCAGCAATAGACATAAGCGGCTATTTAACGACCCTGCCCTGAACCGACGACAAGCTGACGACCGGGTCTCCGCAAGTGGCACTTTTCGGGGAAATGTGCGCGGAACCCCTATTTGTTTATTTTTCTAAATACATTCAAATATGTATCCGCTCATGAATTAATTCTTAGAAAAACTCATCGAGCATCAAATGAAACTGCAATTTATTCATATCAGGATTATCAATACCATATTTTTGAAAAAGCCGTTTCTGTAATGAAGGAGAAAACTCACCGAGGCAGTTCCATAGGATGGCAAGATCCTGGTATCGGTCTGCGATTCCGACTCGTCCAACATCAATACAACCTATTAATTTCCCCTCGTCAAAAATAAGGTTATCAAGTGAGAAATCACCATGAGTGACGACTGAATCCGGTGAGAATGGCAAAAGTTTATGCATTTCTTTCCAGACTTGTTCAACAGGCCAGCCATTACGCTCGTCATCAAAATCACTCGCATCAACCAAACCGTTATTCATTCGTGATTGCGCCTGAGCGAGACGAAATACGCGGTCGCTGTTAAAAGGACAATTACAAACAGGAATCGAATGCAACCGGCGCAGGAACACTGCCAGCGCATCAACAATATTTTCACCTGAATCAGGATATTCTTCTAATACCTGGAATGCTGTTTTCCCGGGGATCGCAGTGGTGAGTAACCATGCATCATCAGGAGTACGGATAAAATGCTTGATGGTCGGAAGAGGCATAAATTCCGTCAGCCAGTTTAGTCTGACCATCTCATCTGTAACATCATTGGCAACGCTACCTTTGCCATGTTTCAGAAACAACTCTGGCGCATCGGGCTTCCCATACAATCGATAGATTGTCGCACCTGATTGCCCGACATTATCGCGAGCCCATTTATACCCATATAAATCAGCATCCATGTTGGAATTTAATCGCGGCCTAGAGCAAGACGTTTCCCGTTGAATATGGCTCATACTCTTCCTTTTTCAATATTATTGAAGCATTTATCAGGGTTATTGTCTCATGAGCGGATACATATTTGAATGTATTTAGAAAAATAAACAAATAGGCATGCAGCGCTCTTCCGCTTCCTCGCTCACTGACTCGCTACGCTCGGTCGTTCGACTGCGGCGAGCGGTGTCAGCTCACTCAAAAGCGGTAATACGGTTATCCACAGAATCAGGGGATAAAGCCGGAAAGAACATGTGAGCAAAAAGCAAAGCACCGGAAGAAGCCAACGCCGCAGGCGTTTTTCCATAGGCTCCGCCCCCCTGACGAGCATCACAAAAATCGACGCTCAAGCCAGAGGTGGCGAAACCCGACAGGACTATAAAGATACCAGGCGTTTCCCCCTGGAAGCTCCCTCGTGCGCTCTCCTGTTCCGACCCTGCCGCTTACCGGATACCTGTCCGCCTTTCTCCCTTCGGGAAGCGTGGCGCTTTCTCATAGCTCACGCTGTTGGTATCTCAGTTCGGTGTAGGTCGTTCGCTCCAAGCTGGGCTGTGTGCACGAACCCCCCGTTCAGCCCGACCGCTGCGCCTTATCCGGTAACTATCGTCTTGAGTCCAACCCGGTAAGACACGACTTATCGCCACTGGCAGCAGCCATTGGTAACTGATTTAGAGGACTTTGTCTTGAAGTTATGCACCTGTTAAGGCTAAACTGAAAGAACAGATTTTGGTGAGTGCGGTCCTCCAACCCACTTACCTTGGTTCAAAGAGTTGGTAGCTCAGCGAACCTTGAGAAAACCACCGTTGGTAGCGGTGGTTTTTCTTTATTTATGAGATGATGAATCAATCGGTCTATCAAGTCAACGAACAGCTATTCCGTTACTCTAGATTTCAGTGCAATTTATCTCTTCAAATGTAGCACCTGAAGTCAGCCCCATACGATATAAGTTGTAATTCTCATGTTAGTCATGCCCCGCGCCCACCGGAAGGAGCTGACTGGGTTGAAGGCTCTCAAGGGCATCGGTCGAGATCCCGGTGCCTAATGAGTGAGCTAACTTACATTAATTGCGTTGCGCTCACTGCCCGCTTTCCAGTCGGGAAACCTGTCGTGCCAGCTGCATTAATGAATCGGCCAACGCGCGGGGAGAGGCGGTTTGCGTATTGGGCGCCAGGGTGGTTTTTCTTTTCACCAGTGAGACGGGCAACAGCTGATTGCCCTTCACCGCCTGGCCCTGAGAGAGTTGCAGCAAGCGGTCCACGCTGGTTTGCCCCAGCAGGCGAAAATCCTGTTTGATGGTGGTTAACGGCGGGATATAACATGAGCTGTCTTCGGTATCGTCGTATCCCACTACCGAGATGTCCGCACCAACGCGCAGCCCGGACTCGGTAATGGCGCGCATTGCGCCCAGCGCCATCTGATCGTTGGCAACCAGCATCGCAGTGGGAACGATGCCCTCATTCAGCATTTGCATGGTTTGTTGAAAACCGGACATGGCACTCCAGTCGCCTTCCCGTTCCGCTATCGGCTGAATTTGATTGCGAGTGAGATATTTATGCCAGCCAGCCAGACGCAGACGCGCCGAGACAGAACTTAATGGGCCCGCTAACAGCGCGATTTGCTGGTGACCCAATGCGACCAGATGCTCCACGCCCAGTCGCGTACCGTCTTCATGGGAGAAAATAATACTGTTGATGGGTGTCTGGTCAGAGACATCAAGAAATAACGCCGGAACATTAGTGCAGGCAGCTTCCACAGCAATGGCATCCTGGTCATCCAGCGGATAGTTAATGATCAGCCCACTGACGCGTTGCGCGAGAAGATTGTGCACCGCCGCTTTACAGGCTTCGACGCCGCTTCGTTCTACCATCGACACCACCACGCTGGCACCCAGTTGATCGGCGCGAGATTTAATCGCCGCGACAATTTGCGACGGCGCGTGCAGGGCCAGACTGGAGGTGGCAACGCCAATCAGCAACGACTGTTTGCCCGCCAGTTGTTGTGCCACGCGGTTGGGAATGTAATTCAGCTCCGCCATCGCCGCTTCCACTTTTTCCCGCGTTTTCGCAGAAACGTGGCTGGCCTGGTTCACCACGCGGGAAACGGTCTGATAAGAGACACCGGCATACTCTGCGACATCGTATAACGTTACTGGTTTCACATTCACCACCCTGAATTGACTCTCTTCCGGGCGCTATCATGCCATACCGCGAAAGGTTTTGCGCCATTCGATGGTGTC

Gray: T7 operator

Purple: *nrk* gene

Orange: T7 terminator

**GSH**

TGGCGAATGGGACGCGCCCTGTAGCGGCGCATTAAGCGCGGCGGGTGTGGTGGTTACGCGCAGCGTGACCGCTACACTTGCCAGCGCCCTAGCGCCCGCTCCTTTCGCTTTCTTCCCTTCCTTTCTCGCCACGTTCGCCGGCTTTCCCCGTCAAGCTCTAAATCGGGGGCTCCCTTTAGGGTTCCGATTTAGTGCTTTACGGCACCTCGACCCCAAAAAACTTGATTAGGGTGATGGTTCACGTAGTGGGCCATCGCCCTGATAGACGGTTTTTCGCCCTTTGACGTTGGAGTCCACGTTCTTTAATAGTGGACTCTTGTTCCAAACTGGAACAACACTCAACCCTATCTCGGTCTATTCTTTTGATTTATAAGGGATTTTGCCGATTTCGGCCTATTGGTTAAAAAATGAGCTGATTTAACAAAAATTTAACGCGAATTTTAACAAAATATTAACGTTTACAATTTCAGGTGGCACTTTTCGGGGAAATGTGCGCGGAACCCCTATTTGTTTATTTTTCTAAATACATTCAAATATGTATCCGCTCATGAATTAATTCTTAGAAAAACTCATCGAGCATCAAATGAAACTGCAATTTATTCATATCAGGATTATCAATACCATATTTTTGAAAAAGCCGTTTCTGTAATGAAGGAGAAAACTCACCGAGGCAGTTCCATAGGATGGCAAGATCCTGGTATCGGTCTGCGATTCCGACTCGTCCAACATCAATACAACCTATTAATTTCCCCTCGTCAAAAATAAGGTTATCAAGTGAGAAATCACCATGAGTGACGACTGAATCCGGTGAGAATGGCAAAAGTTTATGCATTTCTTTCCAGACTTGTTCAACAGGCCAGCCATTACGCTCGTCATCAAAATCACTCGCATCAACCAAACCGTTATTCATTCGTGATTGCGCCTGAGCGAGACGAAATACGCGATCGCTGTTAAAAGGACAATTACAAACAGGAATCGAATGCAACCGGCGCAGGAACACTGCCAGCGCATCAACAATATTTTCACCTGAATCAGGATATTCTTCTAATACCTGGAATGCTGTTTTCCCGGGGATCGCAGTGGTGAGTAACCATGCATCATCAGGAGTACGGATAAAATGCTTGATGGTCGGAAGAGGCATAAATTCCGTCAGCCAGTTTAGTCTGACCATCTCATCTGTAACATCATTGGCAACGCTACCTTTGCCATGTTTCAGAAACAACTCTGGCGCATCGGGCTTCCCATACAATCGATAGATTGTCGCACCTGATTGCCCGACATTATCGCGAGCCCATTTATACCCATATAAATCAGCATCCATGTTGGAATTTAATCGCGGCCTAGAGCAAGACGTTTCCCGTTGAATATGGCTCATAACACCCCTTGTATTACTGTTTATGTAAGCAGACAGTTTTATTGTTCATGACCAAAATCCCTTAACGTGAGTTTTCGTTCCACTGAGCGTCAGACCCCGTAGAAAAGATCAAAGGATCTTCTTGAGATCCTTTTTTTCTGCGCGTAATCTGCTGCTTGCAAACAAAAAAACCACCGCTACCAGCGGTGGTTTGTTTGCCGGATCAAGAGCTACCAACTCTTTTTCCGAAGGTAACTGGCTTCAGCAGAGCGCAGATACCAAATACTGTCCTTCTAGTGTAGCCGTAGTTAGGCCACCACTTCAAGAACTCTGTAGCACCGCCTACATACCTCGCTCTGCTAATCCTGTTACCAGTGGCTGCTGCCAGTGGCGATAAGTCGTGTCTTACCGGGTTGGACTCAAGACGATAGTTACCGGATAAGGCGCAGCGGTCGGGCTGAACGGGGGGTTCGTGCACACAGCCCAGCTTGGAGCGAACGACCTACACCGAACTGAGATACCTACAGCGTGAGCTATGAGAAAGCGCCACGCTTCCCGAAGGGAGAAAGGCGGACAGGTATCCGGTAAGCGGCAGGGTCGGAACAGGAGAGCGCACGAGGGAGCTTCCAGGGGGAAACGCCTGGTATCTTTATAGTCCTGTCGGGTTTCGCCACCTCTGACTTGAGCGTCGATTTTTGTGATGCTCGTCAGGGGGGCGGAGCCTATGGAAAAACGCCAGCAACGCGGCCTTTTTACGGTTCCTGGCCTTTTGCTGGCCTTTTGCTCACATGTTCTTTCCTGCGTTATCCCCTGATTCTGTGGATAACCGTATTACCGCCTTTGAGTGAGCTGATACCGCTCGCCGCAGCCGAACGACCGAGCGCAGCGAGTCAGTGAGCGAGGAAGCGGAAGAGCGCCTGATGCGGTATTTTCTCCTTACGCATCTGTGCGGTATTTCACACCGCATATATGGTGCACTCTCAGTACAATCTGCTCTGATGCCGCATAGTTAAGCCAGTATACACTCCGCTATCGCTACGTGACTGGGTCATGGCTGCGCCCCGACACCCGCCAACACCCGCTGACGCGCCCTGACGGGCTTGTCTGCTCCCGGCATCCGCTTACAGACAAGCTGTGACCGTCTCCGGGAGCTGCATGTGTCAGAGGTTTTCACCGTCATCACCGAAACGCGCGAGGCAGCTGCGGTAAAGCTCATCAGCGTGGTCGTGAAGCGATTCACAGATGTCTGCCTGTTCATCCGCGTCCAGCTCGTTGAGTTTCTCCAGAAGCGTTAATGTCTGGCTTCTGATAAAGCGGGCCATGTTAAGGGCGGTTTTTTCCTGTTTGGTCACTGATGCCTCCGTGTAAGGGGGATTTCTGTTCATGGGGGTAATGATACCGATGAAACGAGAGAGGATGCTCACGATACGGGTTACTGATGATGAACATGCCCGGTTACTGGAACGTTGTGAGGGTAAACAACTGGCGGTATGGATGCGGCGGGACCAGAGAAAAATCACTCAGGGTCAATGCCAGCGCTTCGTTAATACAGATGTAGGTGTTCCACAGGGTAGCCAGCAGCATCCTGCGATGCAGATCCGGAACATAATGGTGCAGGGCGCTGACTTCCGCGTTTCCAGACTTTACGAAACACGGAAACCGAAGACCATTCATGTTGTTGCTCAGGTCGCAGACGTTTTGCAGCAGCAGTCGCTTCACGTTCGCTCGCGTATCGGTGATTCATTCTGCTAACCAGTAAGGCAACCCCGCCAGCCTAGCCGGGTCCTCAACGACAGGAGCACGATCATGCGCACCCGTGGGGCCGCCATGCCGGCGATAATGGCCTGCTTCTCGCCGAAACGTTTGGTGGCGGGACCAGTGACGAAGGCTTGAGCGAGGGCGTGCAAGATTCCGAATACCGCAAGCGACAGGCCGATCATCGTCGCGCTCCAGCGAAAGCGGTCCTCGCCGAAAATGACCCAGAGCGCTGCCGGCACCTGTCCTACGAGTTGCATGATAAAGAAGACAGTCATAAGTGCGGCGACGATAGTCATGCCCCGCGCCCACCGGAAGGAGCTGACTGGGTTGAAGGCTCTCAAGGGCATCGGTCGAGATCCCGGTGCCTAATGAGTGAGCTAACTTACATTAATTGCGTTGCGCTCACTGCCCGCTTTCCAGTCGGGAAACCTGTCGTGCCAGCTGCATTAATGAATCGGCCAACGCGCGGGGAGAGGCGGTTTGCGTATTGGGCGCCAGGGTGGTTTTTCTTTTCACCAGTGAGACGGGCAACAGCTGATTGCCCTTCACCGCCTGGCCCTGAGAGAGTTGCAGCAAGCGGTCCACGCTGGTTTGCCCCAGCAGGCGAAAATCCTGTTTGATGGTGGTTAACGGCGGGATATAACATGAGCTGTCTTCGGTATCGTCGTATCCCACTACCGAGATATCCGCACCAACGCGCAGCCCGGACTCGGTAATGGCGCGCATTGCGCCCAGCGCCATCTGATCGTTGGCAACCAGCATCGCAGTGGGAACGATGCCCTCATTCAGCATTTGCATGGTTTGTTGAAAACCGGACATGGCACTCCAGTCGCCTTCCCGTTCCGCTATCGGCTGAATTTGATTGCGAGTGAGATATTTATGCCAGCCAGCCAGACGCAGACGCGCCGAGACAGAACTTAATGGGCCCGCTAACAGCGCGATTTGCTGGTGACCCAATGCGACCAGATGCTCCACGCCCAGTCGCGTACCGTCTTCATGGGAGAAAATAATACTGTTGATGGGTGTCTGGTCAGAGACATCAAGAAATAACGCCGGAACATTAGTGCAGGCAGCTTCCACAGCAATGGCATCCTGGTCATCCAGCGGATAGTTAATGATCAGCCCACTGACGCGTTGCGCGAGAAGATTGTGCACCGCCGCTTTACAGGCTTCGACGCCGCTTCGTTCTACCATCGACACCACCACGCTGGCACCCAGTTGATCGGCGCGAGATTTAATCGCCGCGACAATTTGCGACGGCGCGTGCAGGGCCAGACTGGAGGTGGCAACGCCAATCAGCAACGACTGTTTGCCCGCCAGTTGTTGTGCCACGCGGTTGGGAATGTAATTCAGCTCCGCCATCGCCGCTTCCACTTTTTCCCGCGTTTTCGCAGAAACGTGGCTGGCCTGGTTCACCACGCGGGAAACGGTCTGATAAGAGACACCGGCATACTCTGCGACATCGTATAACGTTACTGGTTTCACATTCACCACCCTGAATTGACTCTCTTCCGGGCGCTATCATGCCATACCGCGAAAGGTTTTGCGCCATTCGATGGTGTCCGGGATCTCGACGCTCTCCCTTATGCGACTCCTGCATTAGGAAGCAGCCCAGTAGTAGGTTGAGGCCGTTGAGCACCGCCGCCGCAAGGAATGGTGCATGCAAGGAGATGGCGCCCAACAGTCCCCCGGCCACGGGGCCTGCCACCATACCCACGCCGAAACAAGCGCTCATGAGCCCGAAGTGGCGAGCCCGATCTTCCCCATCGGTGATGTCGGCGATATAGGCGCCAGCAACCGCACCTGTGGCGCCGGTGATGCCGGCCACGATGCGTCCGGCGTAGAGGATCGAGATCTCGATCCCGCGAAATTAATACGACTCACTATAGGGGAATTGTGAGCGGATAACAATTCCCCTCTAGAAATAATTTTGTTTAACTTTAAGAAGGAGATATACCATGAAAATAAAAACAGGTGCACGCATCCTCGCATTATCCGCATTAACGACGATGATGTTTTCCGCCTCGGCTCTCGCCAAAATCGAAGAAGGTAAACTGGTAATCTGGATTAACGGCGATAAAGGCTATAACGGTCTCGCTGAAGTCGGTAAGAAATTCGAGAAAGATACCGGAATTAAAGTCACCGTTGAGCATCCGGATAAACTGGAAGAGAAATTCCCACAGGTTGCGGCAACTGGCGATGGCCCTGACATTATCTTCTGGGCACACGACCGCTTTGGTGGCTACGCTCAATCTGGCCTGTTGGCTGAAATCACCCCGGACAAAGCGTTCCAGGACAAGCTGTATCCGTTTACCTGGGATGCCGTACGTTACAACGGCAAGCTGATTGCTTACCCGATCGCTGTTGAAGCGTTATCGCTGATTTATAACAAAGATCTGCTGCCGAACCCGCCAAAAACCTGGGAAGAGATCCCGGCGCTGGATAAAGAACTGAAAGCGAAAGGTAAGAGCGCGCTGATGTTCAACCTGCAAGAACCGTACTTCACCTGGCCGCTGATTGCTGCTGACGGGGGTTATGCGTTCAAGTATGAAAACGGCAAGTACGACATTAAAGACGTGGGCGTGGATAACGCTGGCGCGAAAGCGGGTCTGACCTTCCTGGTTGACCTGATTAAAAACAAACACATGAATGCAGACACCGATTACTCCATCGCAGAAGCTGCCTTTAATAAAGGCGAAACAGCGATGACCATCAACGGCCCGTGGGCATGGTCCAACATCGACACCAGCAAAGTGAATTATGGTGTAACGGTACTGCCGACCTTCAAGGGTCAACCATCCAAACCGTTCGTTGGCGTGCTGAGCGCAGGTATTAACGCCGCCAGTCCGAACAAAGAGCTGGCGAAAGAGTTCCTCGAAAACTATCTGCTGACTGATGAAGGTCTGGAAGCGGTTAATAAAGACAAACCGCTGGGTGCCGTAGCGCTGAAGTCTTACGAGGAAGAGTTGGCGAAAGATCCACGTATTGCCGCCACCATGGAAAACGCCCAGAAAGGTGAAATCATGCCGAACATCCCGCAGATGTCCGCTTTCTGGTATGCCGTGCGTACTGCGGTGATCAACGCCGCCAGCGGTCGTCAGACTGTCGATGAAGCCCTGAAAGACGCGCAGACTCGTATCACCAAGGGCGGAGGCTCCGAGGATCTGTACTTTCAGAGCCATGAATTCATGAGCAAATCTAAAACCTTTCTGTTCACCTCAGAATCTGTAGGTGAAGGTCACCCGGACAAAATCTGCGATCAGGTTAGCGATGCGATCCTGGATGCGTGCCTGGAACAGGATCCGTTCTCTAAAGTTGCGTGCGAAACCGCGGCGAAAACCGGTATGATCATGGTGTTCGGCGAAATTACCACCAAAGCGCGTCTGGATTACCAGCAGATCGTTCGTGACACCATCAAGAAAATCGGCTACGATGACAGCGCGAAAGGTTTCGATTACAAAACCTGCAACGTTCTGGTGGCGATCGAACAGCAGTCTCCGGATATCGCGCAGGGCCTGCATTACGAAAAAAGCCTGGAAGATCTGGGCGCGGGCGATCAGGGTATTATGTTCGGTTACGCGACCGACGAAACCCCGGAAGGCCTGCCGCTGACCATCCTGCTGGCGCACAAACTGAACATGGCGATGGCGGACGCGCGCCGTGACGGCAGCCTGCCGTGGCTGCGCCCGGATACCAAAACCCAGGTTACCGTTGAATACGAAGATGATAACGGCCGTTGGGTGCCGAAACGTATCGATACCGTTGTTATCAGCGCGCAGCACGCGGATGAAATCTCTACCGCGGATCTGCGCACCCAGCTGCAGAAAGATATCGTTGAAAAAGTGATCCCGAAAGATATGCTGGATGAAAACACCAAATACTTCATCCAGCCGTCCGGCCGTTTTGTTATCGGTGGCCCGCAGGGTGACGCGGGTCTGACCGGTCGTAAAATCATCGTTGATGCATACGGCGGCGCGAGCTCCGTTGGTGGTGGCGCTTTCTCCGGTAAAGATTATTCTAAAGTTGATCGTAGCGCGGCATACGCGGCGCGTTGGGTTGCTAAATCCCTGGTTGCTGCGGGTCTGTGTAAACGTGTTCAGGTTCAGTTCTCTTACGCTATCGGTATCGCTGAACCGCTCAGCCTGCACGTTGATACCTATGGCACCGCAACCAAATCTGATGATGAAATCATTGAAATCATTAAAAAGAACTTCGATCTGCGTCCGGGCGTTCTGGTTAAAGAACTGGATCTGGCGCGTCCGATTTACCTGCCGACCGCTTCTTACGGTCACTTCACCAACCAGGAATACTCTTGGGAAAAACCGAAAAAACTGGAATTTTAAAAGCTTCTCGAGCACCACCACCACCACCACTGAGATCCGGCTGCTAACAAAGCCCGAAAGGAAGCTGAGTTGGCTGCTGCCACCGCTGAGCAATAACTAGCATAACCCCTTGGGGCCTCTAAACGGGTCTTGAGGGGTTTTTTGCTGAAAGGAGGAACTATATCCGGATTAATACGACTCACTATAGG

Gray: T7 operator

Green: *sam2* gene

Orange: T7 terminator

**HIS**

GATACGAGAGACCTCTCTTGTATCTTTTTTATTTTGAGTGGTTTTGTCCGTTACACTAGAAAACCGAAAGACAATAAAAATTTTATTCTTGCTGAGTCTGGCTTTCGGTAAGCTAGACAAAACGGACAAAATAAAAATTGGCAAGGGTTTAAAGGTGGAGATTTTTTGAGTGATCTTCTCAAAAAATACTACCTGTCCCTTGCTGATTTTTAAACGAGCACGAGAGCAAAACCCCCCTTTGCTGAGGTGGCAGAGGGCAGGTTTTTTTGTTTCTTTTTTCTCGTAAAAAAAAGAAAGGTCTTAAAGGTTTTATGGTTTTGGTCGGCACTGCCGACAGCCTCGCAGAGCACACACTTTATGAATATAAAGTATAGTGTGTTATACTTTACTTGGAAGTGGTTGCCGGAAAGAGCGAAAATGCCTCACATTTGTGCCACCTAAAAAGGAGCGATTTACATATGATGAAAACAGAAAGAGAAAACGGAATCGTCCGTCAAGTGAATACGATTCAAACAAAAGAAGAGCGCTTTAATCCTTTCTCTTGGTACGAAGAGATGAGAAACAGTGAACCTGTGCAGTGGGATGAAGAAAGGCAGGTATGGGATGTTTTTCACTATGACGGAGTCAAAGAAGTACTGGAGCAAAAGAATATTTTTTCTTCTGATCGAAGACCTCCACAAAACCAAAGACAAACTGCTTTAGGAACGAGCCTAATTAATATTGATCCGCCTAAGCACGCTGAAATGAGAGCACTTGTTAATAAAGCTTTTACGCCTAAAGCAATGAAAGCATGGGAGCCTAAAATTGCTCGCATTACACATGAATTATTACAAGAAGTTGAGCACCTTGAAGACATTGATATAGTCGAGCATCTTTCCTACCCGCTTCCGGTTATGGTGATTGCCGATATATTAGGCGTGCCGATAGAAGACCAGCGTCAGTTTAAAGATTGGTCGGATATTATCGTAGCGGGTCCGTCGAATAATGAACGTGAAACGCTAGAAAAATTGCAGCAAGAGAAAATGAAGGCAAATGATGAGCTAGAAACTTACTTTTATCGAATCATTGAAGAAAAACGCACCCATCCAGGAGATGATATTATCTCCGTGCTTCTTCAGGCAAAAGAAGAAGGGACACAGCTAACGGATGAAGAAATCGTAGGGTTTTCCATTTTGCTGTTGATTGCAGGAAACGAAACCACAACAAATTTAATTTCAAATACGATTTATTGTTTAATGGAAGATAAAGCTTCTTTTGAACGACTCAAACGAGAGAAAGAACTTTTGCCTTCTGCGATTGAAGAAGTTCTTCGCTATCGTTCACCCGTTCAAGCTCTTCACCGAATCGTAAAAGAAGATGTGATTCTTGCAGGCAAGAAATTAAAAGCAGGAGAACACGTCATTCCATGGATGGGATCAGCGCACCGAGACGCGCAGTACTTTGAAGACCCGGATGTATTTAAAATCGACCGAAAGCCAAATATGCATATGGCATTTGGAAGAGGCATTCATTTTTGCTTAGGAGCACCGCTTGCTCGAATAGAAGCAAAAATTATGCTATCTGAGCTGATCGACCGCTATCCGCATATGGACTGGAGCCCGTCATTTGAGTTAAAGCCGATTGAAAGCACATTTGTGTATGGGTTAAAAGAATTATTGATTCGTAAAAACGTACATCATCATCATCATCATTAAGGATCCTCTAGAGTCGAGCTCAAGCTAGCTTGGTACGTACCAGATCTGAGATCACGCGTTCTAGAGGTCGAAATTCACCTCGAAAGCAAGCTGATAAACCGATACAATTAAAGGCTCCTTTTGGAGCCTTTTTTTTTGGAGATTTTCAACGTGAAAAAATTATTATTCGCAATTCCAAGCTAATTCACCTCGAAAGCAAGCTGATAAACCGATACAATTAAAGGCTCCTTTTGGAGCCTTTTTTTTTGGAGATTTTCAACGTGAAAAAATTATTATTCGCAATTCCAAGCTCTGCCTCGCGCGTTTCGGTGATGACGGTGAAAACCTCTGACACATGCAGCTCCCGGAGACGGTCACAGCTTGTCTGTAAGCGGATGCAGATCACGCGCCCTGTAGCGGCGCATTAAGCGCGGCGGGTGTGGTGGTTACGCGCAGCGTGACCGCTACACTTGCCAGCGCCCTAGCGCCCGCTCCTTTCGCTTTCTTCCCTTCCTTTCTCGCCACGTTCGCCGGCTTTCCCCGTCAAGCTCTAAATCGGGGGCTCCCTTTAGGGTTCCGATTTAGTGCTTTACGGCACCTCGACCCCAAAAAACTTGATTAGGGTGATGGTTCACGTAGTGGGCCATCGCCCTGATAGACGGTTTTTCGCCCTTTGACGTTGGAGTCCACGTTCTTTAATAGTGGACTCTTGTTCCAAACTGGAACAACACTCAACCCTATCTCGGTCTATTCTTTTGATTTATAAGGGATTTTGCCGATTTCGGCCTATTGGTTAAAAAATGAGCTGATTTAACAAAAATTTAACGCGAATTTTAACAAAATATTAACGCTTACAATTTGATCTGCGCTCGGTCGTTCGGCTGCGGCGAGCGGTATCAGCTCACTCAAAGCGGTAATACGGTTATCCCAGAATCCGGGGATAACGCAGGAAAGACAGGTGGCACTTTTCGGGGAAATGTGCGCGGAACCCCTATTTGTTTATTTTTCTAAATACATTCAAATATGTATCCGCTCATGAGACAATAACCCTGATAAATGCTTCAATAATATTGAAAAAGGAAGAGTATGAGTATTCAACATTTCCGTGTCGCCCTTATTCCCTTTTTTGCGGCATTTTGCCTTCCTGTTTTTGCTCACCCAGAAACGCTGGTGAAAGTAAAAGATGCTGAAGATCAGTTGGGTGCACGAGTGGGTTACATCGAACTGGATCTCAACAGCGGTAAGATCCTTGAGAGTTTTCGCCCCGAAGAACGTTTTCCAATGATGAGCACTTTTAAAGTTCTGCTATGTGGCGCGGTATTATCCCGTGTTGACGCCGGGCAAGAGCAACTCGGTCGCCGCATACACTATTCTCAGAATGACTTGGTTGAGTACTCACCAGTCACAGAAAAGCATCTTACGGATGGCATGACAGTAAGAGAATTATGCAGTGCTGCCATAACCATGAGTGATAACACTGCGGCCAACTTACTTCTGACAACGATCGGAGGACCGAAGGAGCTAACCGCTTTTTTGCACAACATGGGGGATCATGTAACTCGCCTTGATCGTTGGGAACCGGAGCTGAATGAAGCCATACCAAACGACGAGCGTGACACCACGATGCCTGCAGCAATGGCAACAACGTTGCGCAAACTATTAACTGGCGAACTACTTACTCTAGCTTCCCGGCAACAATTAATAGACTGGATGGAGGCGGATAAAGTTGCAGGACCACTTCTGCGCTCGGCCCTTCCGGCTGGCTGGTTTATTGCTGATAAATCTGGAGCCGGTGAGCGTGGGTCTCGCGGTATCATTGCAGCACTGGGGCCAGATGGTAAGCCCTCCCGTATCGTAGTTATCTACACGACGGGGAGTCAGGCAACTATGGATGAACGAAATAGACAGATCGCTGAGATAGGTGCCTCACTGATTAAGCATTGGTAACTGTCAGACCAAGTTTACTCATATATACTTTAGATTGATTTAAAACTTCATTTTTAATTTAAAAGGATCTAGGTGAAGATCCTTTTTGATAATCTCATGACCAAAATCCCTTAACGTGAGTTTTCGTTCCACTGAGCGTCAGACCCCGTAGAAAAGATCAAAGGATCTTCTTGAGATCCTTTTTTTCTGCGCGTAATCTGCTGCTTGCAAACAAAAAAACCACCGCTACCAGCGGTGGTTTGTTTGCCGGATCAAGAGCTACCAACTCTTTTTCCGAAGGTAACTGGCTTCAGCAGAGCGCAGATACCAAATACTGTCCTTCTAGTGTAGCCGTAGTTAGGCCACCACTTCAAGAACTCTGTAGCACCGCCTACATACCTCGCTCTGCTAATCCTGTTACCAGTGGCTGCTGCCAGTGGCGATAAGTCGTGTCTTACCGGGTTGGACTCAAGACGATAGTTACCGGATAAGGCGCAGCGGTCGGGCTGAACGGGGGGTTCGTGCACACAGCCCAGCTTGGAGCGAACGACCTACACCGAAGTACTGAGATACCTACAGCGTGAGCTATGAGAAAGCGCCACGCTTCCCGAAGGGAGAAAGGCGGACAGGTATCCGGTAAGCGGCAGGGTCGGAACAGGAGAGCGCACGAGGGAGCTTCCAGGGGGAAACGCCTGGTATCTTTATAGTCCTGTCGGGTTTCGCCACCTCTGACTTGAGCGTCGATTTTTGTGATGCTCGTCAGGGGGGCGGAGCCTATGGAAAAACGCCAGCAACGCGGCCTTTTTACGGTTCCTGGCCTTTTGCTGGCCTTTTGCTCACATGTTCTTTCCTGCGTTATCCCCTGATTCTGTGGATAACCGTATTACCGCCTTTGAGTGAGCTGATACCGCTCGCCGCAGCCGAACGACCGAGCGCAGCGAGTCAGTGAGCGAGGAAGCGGAAGAGCGCCCAATACGCAAACCGCCTCTCCCCGCGCGTTGGCCGATTCATTAATGCAGCTGGCACGACAGGTTTCCCGACTGGAAAGCGGGCAGTGAGCGCAACGCAATTAATGTGAGTTAGCTCACTCATTAGGCACCCCAGGCTTTACACTTTATGCTTCCGGCATATTCTCAATAAACCCTTTAGGGAAATAGGCCAGGTTTTCACCGTAACACGCCACATCTTGCGAATATATGTGTAGAAACTGCCGGAAATCGTCGTGGTATTCACTCCAGAGCGATGAAAACGTTTCAGTTTGCTCATGGAAAACGGTGTAACAAGGGTGAACACTATCCCATATCACCAGCTCACCGTCTTTCATTGCCATACGAAATTCCGGATGAGCATTCATCAGGCGGGCAAGAATGTGAATAAAGGCCGGATAAAACTTGTGCTTATTTTTCTTTACGGTCTTTAAAAAGGCCGTAATATCCAGCTAAACGGTCTGGTTATAGGTACATTGAGCAACTGACTGAAATGCCTCAAAATGTTCTTTACGATGCCATTGGGATATATCAACGGTGGTATATCCAGTGATTTTTTTCTCCATTTTAGCTTCCTTAGCTCCTGAAAATCTCGATAACTCAAAAAATACGCCCGGTAGTGATCTTATTTCATTATGGTGAAAGTTGGAACCTCTTACGTGCCGATCAA

Gray: HpaⅡ operator

Blue: *cyp109e1* gene

Orange: fd terminator
